# Transcriptional signature of durable effector T cells elicited by a replication defective HCMV vaccine

**DOI:** 10.1038/s41541-024-00860-w

**Published:** 2024-04-01

**Authors:** Xiaohua Ye, David J. H. Shih, Zhiqiang Ku, Junping Hong, Diane F. Barrett, Richard E. Rupp, Ningyan Zhang, Tong-Ming Fu, W. Jim Zheng, Zhiqiang An

**Affiliations:** 1 Texas Therapeutics Institute, Brown Foundation Institute of Molecular Medicine, University of Texas Health Science Center at Houston, Houston, TX USA; 2 School of Biomedical Informatics, University of Texas Health Science Center at Houston, Houston, TX USA; 3 Sealy Institute for Vaccine Sciences, University of Texas Medical Branch, Galveston, TX USA; 4 Present Address: Center for Infectious Disease Research, School of Life Sciences, Westlake University, Hangzhou, Zhejiang China; 5 Present Address: School of Biomedical Sciences, The University of Hong Kong, Pokfulam, Hong Kong SAR

**Keywords:** Live attenuated vaccines, Viral infection, Immunological memory

## Abstract

Human cytomegalovirus (HCMV) is a leading infectious cause of birth defects and the most common opportunistic infection that causes life-threatening diseases post-transplantation; however, an effective vaccine remains elusive. V160 is a live-attenuated replication defective HCMV vaccine that showed a 42.4% efficacy against primary HCMV infection among seronegative women in a phase 2b clinical trial. Here, we integrated the multicolor flow cytometry, longitudinal T cell receptor (TCR) sequencing, and single-cell RNA/TCR sequencing approaches to characterize the magnitude, phenotype, and functional quality of human T cell responses to V160. We demonstrated that V160 de novo induces IE-1 and pp65 specific durable polyfunctional effector CD8 T cells that are comparable to those induced by natural HCMV infection. We identified a variety of V160-responsive T cell clones which exhibit distinctive “transient” and “durable” expansion kinetics, and revealed a transcriptional signature that marks durable CD8 T cells post-vaccination. Our study enhances the understanding of human T-cell immune responses to V160 vaccination.

## Introduction

Human cytomegalovirus (HCMV) is a ubiquitous β-herpesvirus that establishes life-long latent infection^1^. HCMV is an important cause of mortality and morbidity among transplant patients^2^, and congenital HCMV infection is the leading cause of infectious childhood disability^3^. For these reasons, HCMV has been a high priority for vaccine development^4^. Different kinds of vaccines, including live-attenuated viruses, glycoprotein subunit formulations, viral vectors, single/bivalent DNA plasmids, and RNA vaccines, have been developed and evaluated to prevent HCMV infections and associated morbidities since the 1970s^5^. None has been successful yet.

V160 is a single-cycle and replication-defective whole virus vaccine based on the attenuated HCMV strain AD169^6^. There were two modifications at the genetic level to engineer AD169 into V160. One modification restores expression of the epithelial/endothelial tropism determinant known as gH/gL/pUL128/130/131 complex or the pentamer. The other modification fuses two essential viral proteins (IE1/2 and pUL51) to the degradation domain FKBP (ddFKBP), which mediates the default degradation of newly synthesized fusion proteins in the absence of a small molecule compound Shield-1^6^. In a phase 1 clinical study (NCT01986010), human subjects vaccinated with V160 were without signs of HCMV infection and viral shedding, confirming that the virus is replication defective in vivo. Additionally, the vaccine appeared to be safe and well tolerated^7^. A double-blind multicenter phase 2b clinical study evaluated the efficacy of V160 to prevent HCMV infection (NCT03486834) among healthy seronegative women 16–35 years of age. Vaccine efficacy was 42.4% in the 3-dose V160 group versus placebo against wild-type HCMV infection, and both the quantity and duration of viral shedding in urine and saliva among infected subjects were reduced in the 3-dose group^8,9^. The gB/MF59 subunit vaccine provided 50% protection against HCMV infection in seronegative postpartum women^10^ and 43% protection in seronegative adolescent girls^11^. The Triplex vaccine is a modified vaccine Ankara (MVA) encoding HCMV pp65, IE1-exon4, and IE2-exon5. Triplex vaccination of patients after hematopoietic stem cell transplant (HSCT) reduced the risk for a significant HCMV event by half during the first 100 days after transplant^12^. Although the efficacy of the 3-dose V160 does not reach a conventional significant level, it is one of the most successful vaccines for HCMV prevention to date.

Natural HCMV infection elicits potent humoral and cellular immune responses for immune control of latent HCMV infection. The immune correlates of protection against HCMV acquisition, replication, and disease are likely a combination of innate immune factors, antibodies, and T-cell responses^13^. An ideal HCMV vaccine would probably need to elicit a strong and balanced immune response targeting multiple viral antigens. Reinfection or reactivation does occur during pregnancy, but the rate of congenital HCMV is lower in women with non-primary infection than with primary infection^14,15^. Protection against congenital transmission in women with primary HCMV infection is associated with early emergence of CD4 T cells^16^. The importance of T cell immunity in the control of HCMV in transplant patients has long been recognized^17^. Notably, the total number of HCMV-specific T cells is enormous in seropositive individuals, comprising, on average, ~10% of both the CD4 and CD8 T cell memory compartments in blood^18^. HCMV-induced CD8 T cells are characterized by the accumulation of terminally differentiated effector memory T cells re-expressing CD45RA (the T_EMRA_ subset), which persist lifelong during latent phase^19^.

As a replication-defective vaccine, V160 showed encouraging results with regard to the elicitation of humoral immunity. The levels of V160-induced neutralizing antibodies in HCMV-seronegative subjects in a phase 1 study were within ranges of natural HCMV-infected individuals. Further, the quality of V160-induced antibody responses was analogous to those induced by natural HCMV infections at the level of monoclonal antibodies^20–22^. V160 elicited T-cell responses were evaluated in a small group of subjects of a phase 1 study, which reveals a genetically diverse and polyfunctional T-cell response against IE-1 and pp65^7,23^. In this study, we integrated multicolor flow cytometry, single-cell RNA sequencing (scRNA-seq), single-cell T cell receptor sequencing (scTCR-seq), and longitudinal blood TCR expansion analysis to further characterize V160-induced T cell responses in additional HCMV-seronegative subjects from a phase 2 trial of V160. We confirmed that V160 de novo induces potent IE-1 and pp65 specific polyfunctional CD4 T and CD8 T cell responses, which are similar to those induced by natural HCMV infection. We identified V160-responsive T-cell clones with distinctive expansion kinetics and analyzed their unique transcriptome features. Our study enhances the understanding of human T-cell responses to V160 vaccination and underscores the promise of replication of defective HCMV as a vector for vaccine development.

## Results

### V160 elicits polyfunctional T-cell responses in HCMV-seronegative subjects

**Fig. 1:**
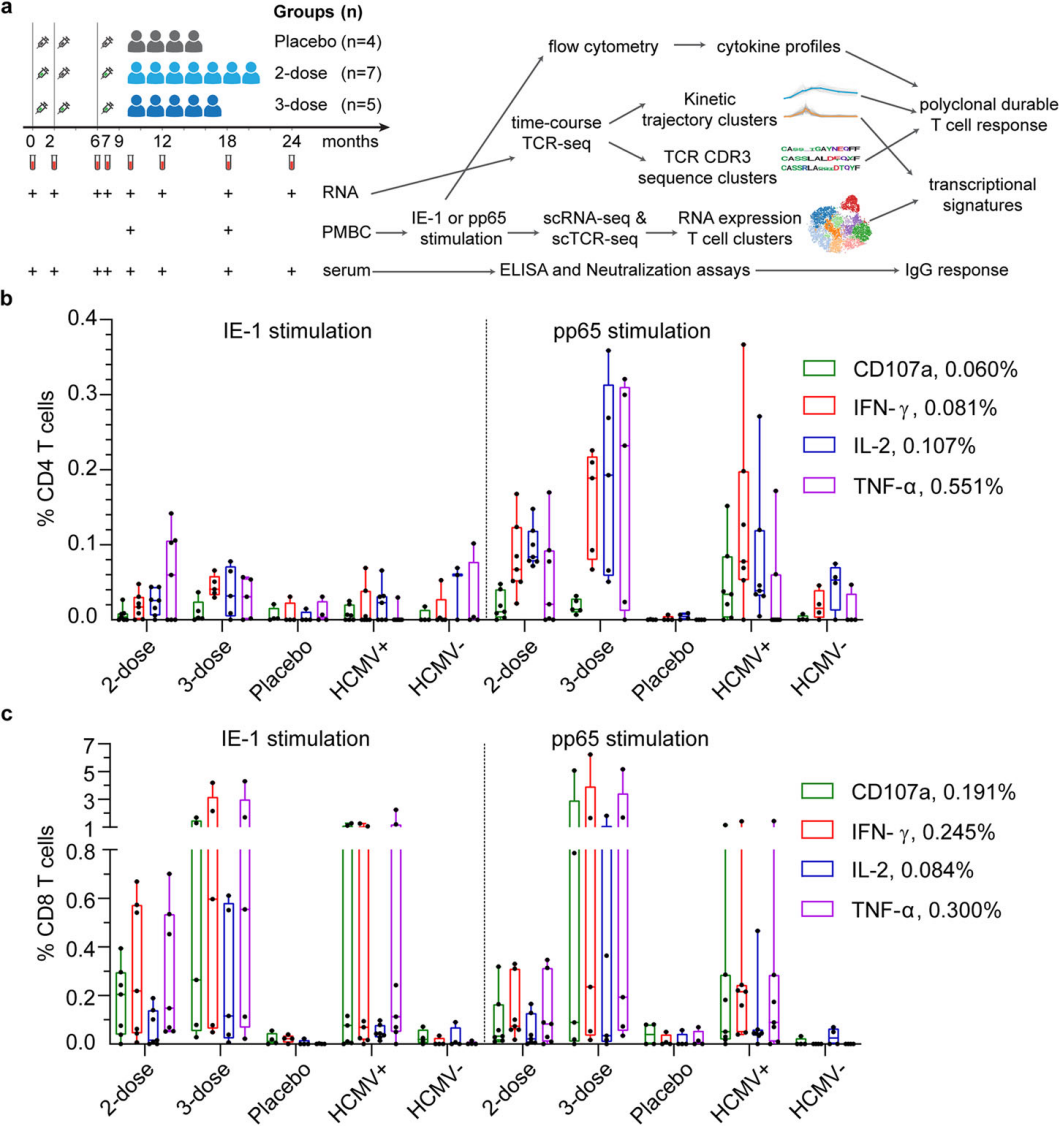
V160 induced IE-1 and pp65 T-cell responses as compared to natural HCMV infection. **a** Overview of study design, sample collection, experiments, and analyses. Month 9 PBMCs of the 2-dose group (*n* = 7), 3-dose group (*n* = 5), and placebo group (*n* = 4) together with PBMCs of HCMV^+^ donors (*n* = 7) and HCMV^−^ donors (*n* = 4) were analyzed for T-cell responses to IE-1 and pp65 by intracellular cytokine staining (ICS) flow cytometry. **b** and **c** Percentages of (**b**) CD4 T cells and (**c**) CD8 T cells that expressed four kinds of effector molecules (CD107a, IFN-γ, IL-2, and TNF-α) after DMSO background subtraction (supplementary data file 1). Data are plotted in a box and whiskers style showing the median (center line), the first quartile, and the third quartile together with all data points. Black dots indicate individual responses. Positive cutoffs of specific T-cells were calculated from the median plus a two-fold standard error of the mean of background responses in all individuals (Supplementary Fig. 2) and were shown next to the corresponding legend.

To characterize T cell responses induced by V160 vaccination, we collected blood samples for isolation of RNA, PBMCs, and serum samples from subjects enrolled at one site participating in the V160 phase 2b clinical trial (NCT03486834). One hundred units of V160 or an equal volume of saline solution containing the Merck aluminum phosphate adjuvant (MAPA) was administered as a 0.5 ml intramuscular injection. HCMV-seronegative subjects received intramuscular injections of two doses of V160 plus one dose of placebo (2-dose group), three doses of V160 (3-dose group), or three doses of placebo (placebo group) at day 1, month 2, and month 6 as indicated (Fig. 1a). We detected HCMV IE-1 and pp65 specific T cells in peripheral blood mononuclear cells (PBMCs) collected from V160 and placebo subjects at month 9, and included PBMCs from age- and sex-matched healthy HCMV seropositive (HCMV^+^) and seronegative (HCMV^−^) blood donors as controls. Following stimulation by 15-mer peptide pools of IE-1, pp65, or DMSO control (peptide solvent) together with co-stimulators, the PBMCs were analyzed by intracellular cytokines staining (ICS) flow cytometry combined with cell surface markers staining for T cell differentiation status. Sequential gating strategies were used for the analysis of responding CD4 and CD8 T cells that express four effector molecules (IFN-γ, IL-2, TNF-α, and CD107a) (Supplementary Fig. 1). The level of DMSO background responses was shown in Supplementary Fig. 2. Antigen-specific CD4 T and CD8 T cells that expressed four effector molecules were determined as DMSO background subtracted data (Fig. 1b, c). The positive cutoffs were calculated from the median plus two-fold standard error of the mean of the DMSO background responses in 27 analyzed individuals. A positive response is defined as higher than corresponding positive cutoffs. A positive responder is defined as an individual with at least one positive response. Based on the positive cutoffs, there was one marginal positive response among four HCMV^−^ individuals, but no positive response was observed in the placebo group (0/4). About 71.4% (5/7) subjects in the 2-dose group, 80% (4/5) subjects in the 3-dose group, and 57.1% (4/7) donors in the HCMV^+^ group were positive T-cell responders (supplementary data file 1). Overall, higher percentages of antigen-specific CD8 T cells are detected than antigen-specific CD4 T cells for both the V160 subjects and HCMV^+^ donors, and the percentages of pp65-specific CD4 T cells are higher than that of IE-1-specific CD4 T cells, which are below positive cutoffs in all individuals (Fig. 1b, c). Due to a small number of subjects and big variations in T-cell responses, our data lack the power for reliable comparisons between 2-dose and 3-dose groups. Notably, two subjects (S26 and S28) with the most potent CD8 T-cell responses (up to 6.2% IFN-γ^+^ CD8 T cells) have both received 3 doses V160 (Supplementary Fig. 3). In addition, all V160-vaccinated subjects showed strong serum antibody responses to multiple HCMV antigens (including gB, pentamer and whole virion), as well as high serum neutralizing titers against HCMV infection in ARPE-19 cells (Supplementary Fig. 4), including three V160 subjects who had negative T-cell response to both IE-1 and pp65 stimulations according to our positive cutoffs. These results demonstrate that V160 vaccination of seronegative subjects efficiently induces HCMV-specific antibody responses among all subjects while the sizes of T-cell responses are heterogeneous among the subjects.

**Fig. 2:**
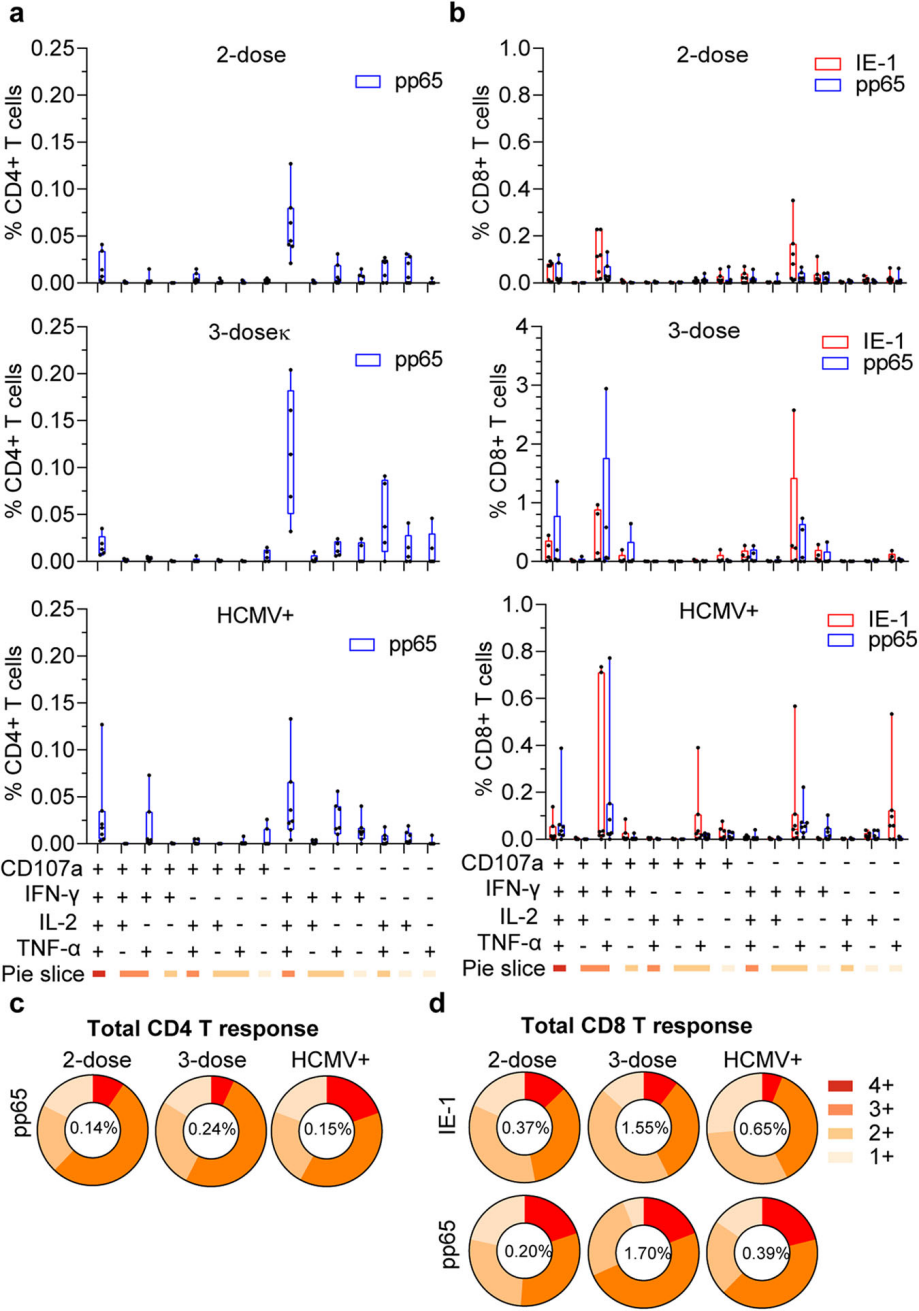
Polyfunctional status of IE-1 and pp65 specific T cells induced by V160 and HCMV infection. Combinatorial analysis of IE-1 and pp65 responding T cells in Fig. 1 was performed. **a** and **b** The percentages of (**a**) CD4 and (**b**) CD8 T cells that expressed fifteen combinations of four effector molecules (CD107a, IFN-γ, IL-2, and TNF-α) after background subtraction. Data were shown in a box and whiskers style showing the median (center line), the first quartile, and the third quartile together with all data points. Black dots indicate individual responses. **c** and **d** Average proportions of antigen-specific (**c**) CD4 or (**d**) CD8 T cells in each group that were positive for 1–4 effectors. The average total responsive T cells in each group were shown in the center of the pie chart. Individual data of Fig. 2c, d are shown in Supplementary Fig. 5.

To compare the quality of T cell responses in month 9 PBMCs of V160-vaccinated subjects and HCMV^+^ donors, we further determined the percentages of T cells that co-expressed each combination of four effector molecules (Fig. 2). Since the IE-1 responding CD4 T cells were below positive cutoffs in all analyzed individuals, we did not include it in the analysis. The overall results of 2-dose and 3-dose V160 subjects were very similar to those of HCMV^+^ donors. Specifically, pp65 responding CD4 T cells had a dominant CD107a^−^IFN-γ^+^IL-2^+^TNF-α^+^ subset, while IE-1 and pp65 responding CD8 T cells had two dominant subsets, the CD107a^+^IFN-γ^+^IL-2^−^TNF-α^+^ and CD107a^−^IFN-γ^+^IL-2^−^TNF-α^+^ subsets (Fig. 2a, b). Average proportions of responding T cells that were positive for one, two, three, or four effector molecules in each group are shown as pie charts, with the average total frequency of responding T cells indicated in the center (Fig. 2c, d, Supplementary Fig 5). Significant proportions of responding CD4 T cells and CD8 T cells co-expressed four effector molecules, and ~80% of total responding cells co-expressed ≥2 effector molecules in all three groups. Taken together, the V160-induced IE-1- and pp65-specific polyfunctional T-cell responses are similar to those induced by natural HCMV infection.

### Single-cell transcriptomics of CD3^+^ T cells after in vitro stimulation

**Fig. 3:**
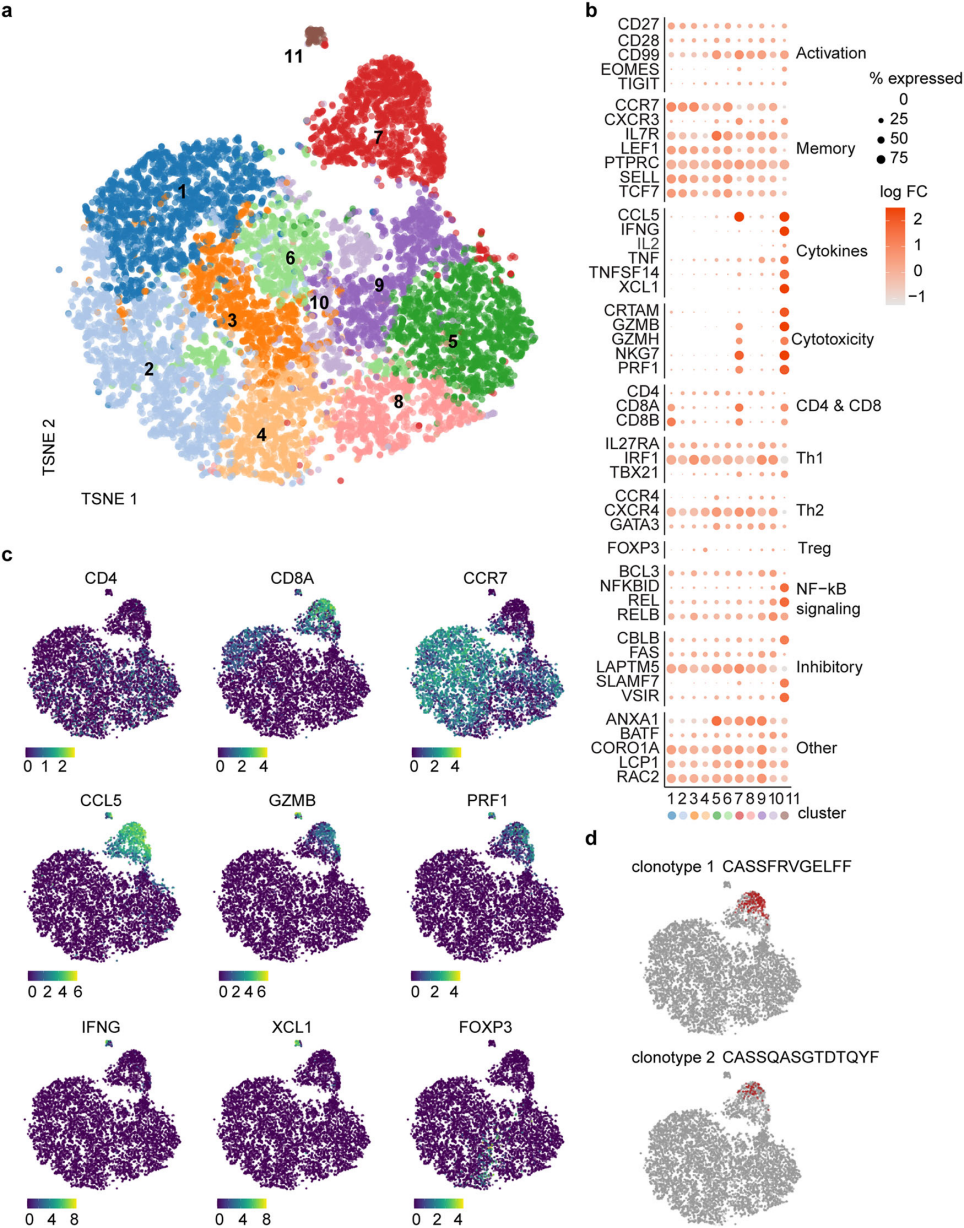
Transcriptional makeup of CD3^+^ T cells post V160 vaccination. **a** Single-cell transcriptomes of enriched CD3^+^ T cells from IE-1 and pp65 stimulated month 18 PBMC of S26 were shown as a t-distributed stochastic neighbor embedding (TSNE) plot, colored based on RNA expression cluster assignment by the Louvain graph-based method. **b** Percentage of cells expressing selected genes within each expression cluster (size scale) and log fold change (FC) of expression within each cluster vs. other clusters (color scale). **c** Expression levels of selected genes are shown as color overlays on the TSNE plot from (**a**). **d** Highlighting of T cells of the two most prevalent clonotypes (with CDR3β sequences shown) on the TSNE plot.

To better understand the long-term T cell response after V160 vaccination, we performed single-cell transcriptomic characterization and paired T cell receptor (TCR) α/β analysis using the 10× Genomics Chromium platform in subject S26, who received 3 doses of V160 and showed very strong T-cell response. We stimulated month 18 PMBCs of S26 (one year post the last vaccination) with peptide pools of IE-1 and pp65; we then sorted the live CD3^+^ T cells without antigen-specific enrichment and performed scRNA-seq analysis. Unsupervised clustering of the single-cell expression data segregated the T cells into three main CD8 T cell clusters (clusters 1, 7, and 11) and several CD4 clusters (Fig. 3a–c). Cluster 7 and 11 express many cytotoxic effectors such as granzyme B (GZMB), granzyme H (GZMH), natural killer cell granule protein 7 (NKG7), and perforin 1 (PRF1), while C–C motif chemokine receptor 7 (CCR7) expression is low in these clusters (Fig. 3b, c). Additionally, cytokines such as interferon-gamma (IFNG) and X-C motif chemokine ligand 1 (XCL1) are most highly expressed only in cluster 11. Regulatory molecules such as CBLB, an E3 ubiquitin-protein ligase, and the V-set immunoregulatory receptor (VSIR, also known as VISTR) are also most frequently expressed in cluster 11 (Fig. 3b). Conversely, CD4 T cell clusters are highly heterogeneous (Fig. 3b, c). Only two TCR clonotypes have a frequency > 0.5%, which is consistent with the absence of specific T-cell enrichment. These two clonotypes are both located in the cytotoxic CD8 cluster 7 (Fig. 3d). Further analysis is needed to locate V160-responsive T cells among different clusters.

### Longitudinal TCR profiling identifies V160 responsive T cell clonotypes

**Fig. 4:**
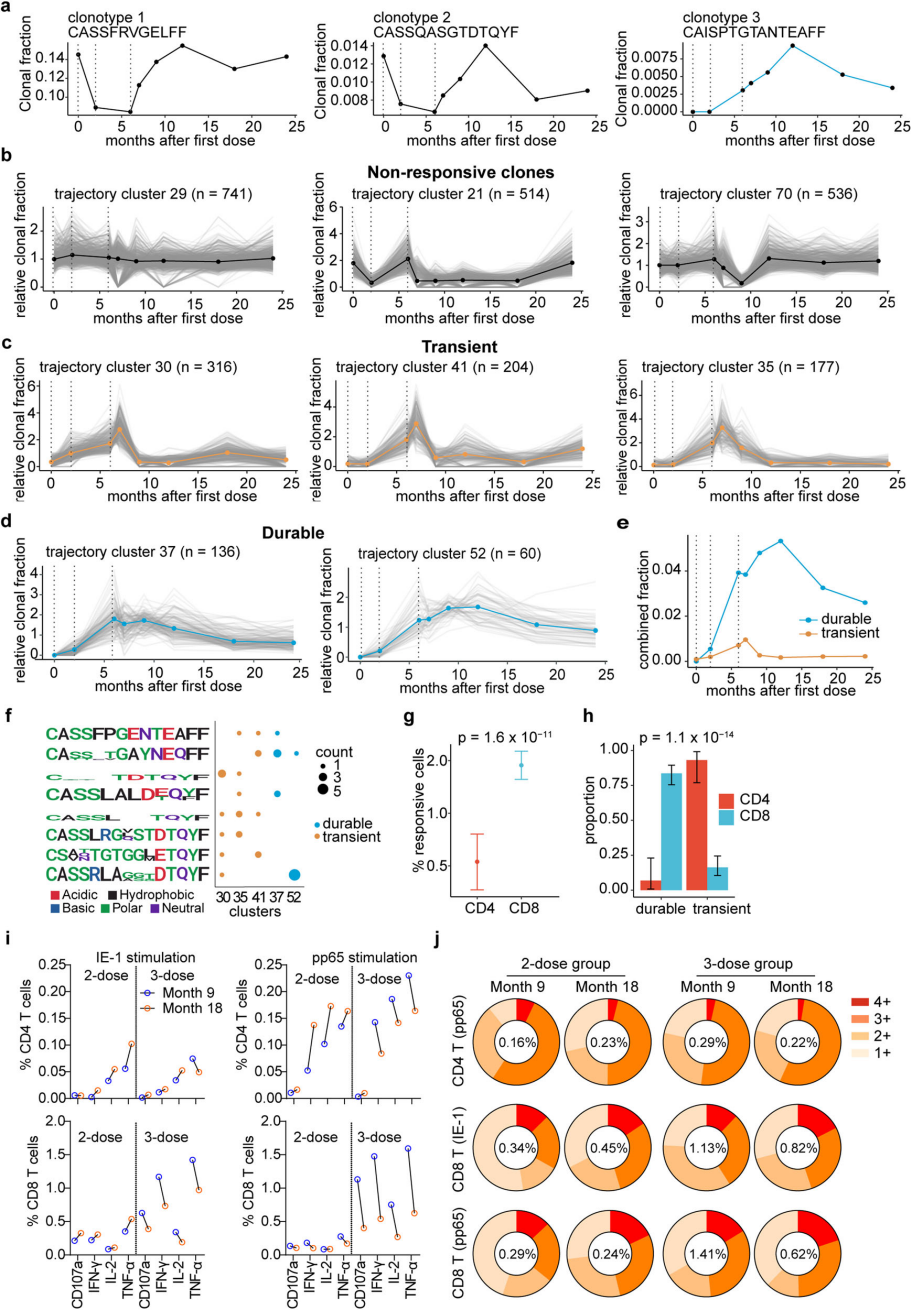
V160 elicits durable polyclonal CD8 T cell response. **a–e** Time-course blood TCR profiling of a 3-dose V160 subject (S26) at eight time points with dotted lines indicating V160 injections (D1, M2, and M6). **a** Clonal fractions of three most prevalent clones are shown with CDR3β sequences. **b–d** Trajectory clustering of time-course TCR profiles. **b** Three representative clusters of non-responsive T cell clones. Clusters of T cell clones exhibiting (**c**) “transient” or (**d**) “durable” clonal expansion in response to V160 vaccination. *n* denotes the number of unique T cell clones. **e** Combined clonal fractions of durable and transient T cell clones. **f** TCR CDR3β sequence clusters are shown as sequence logos, with the number of T clones assigned to each CDR3β sequence cluster and each trajectory cluster shown as a dot plot. **g** Percentages of V160-responsive CD4 or CD8 T cells in scRNA-seq/scTCR-seq data were identified using the CDR3β as a natural barcode. Significance was assessed by Fisher’s exact test. **h** Proportions of V160-responsive CD4 and CD8 T cells that were “durable” and “transient” clones. Significance was assessed by Fisher’s exact test. Error bars in **g**, **h** indicate mean ± standard error of the mean. **i** Average percentages of IE-1 or pp65 specific CD4 or CD8 T cells in month 9 (blue circle) or 18 PBMCs (orange circle) from 2-dose (*n* = 5) and 3-dose (*n* = 5) subjects were compared side-by-side for expression of four effector molecules (CD107a, IFN-γ, IL-2, and TNF-α) by ICS flow cytometry. Data were background subtracted. Individual responses in **i** are shown in Supplementary Fig. 6. **j** Distributions of responsive CD4 vs. CD8 T cells positive for 1, 2, 3, or 4 of the four effector molecules in month 9 vs. 18 PBMCs following IE-1 or pp65 antigen stimulation. The average percentage of total responsive T cells in each group was shown in the center of the pie chart. Data were background subtracted. Individual responses in **j** are shown in Supplementary Fig. 8.

To track T-cell clonal expansion post V160 vaccination, we performed longitudinal blood TCR CDR3β sequencing to samples from the same subject (S26) at 8-time points up to 24 months. We identified 1,936,450 distinct TCR clonotypes from a total of 2,318,537 detected TCR CDR3β sequences. A total of 33,074 unique clones were supported by ≥2 reads in ≥3 samples (time points). The two most prevalent clonotypes (clonotype 1 and 2) represented up to 15% and 1.5% of TCR sequences, and their CDR3β sequences (CASSFRVGELFF and CASSQASGTDTQYF) matched the same dominant clonotypes identified in the single cell paired TCR α/β analysis at month 18 (Fig. 3d). Surprisingly, the clonal fractions of these two clonotypes showed high-level baselines at day 1 and declined at months 2 and 6 after the first and second dose V160 vaccination, which indicates these dominant clonotypes were not induced by V160 (Fig. 4a). In contrast, the clonal fraction of clonotype 3 was undetected at baseline (day 1) and showed a consistent increase after each dose of V160 and peaked at month 12 (Fig. 4a), which suggests that this clonotype underwent V160-induced clonal expansion. To identify other clonotypes that respond similarly to V160 vaccination, we performed unsupervised *K*-means clustering (*K* = 100) of the longitudinal relative clonal fraction changes of 33,074 unique CDR3β clonotypes at eight-time points. The clusters containing TCR clones that are consistently expanded post each vaccination (at months 2, 6, and 7) were considered V160 responsive. Most trajectory clusters appeared unresponsive to V160 vaccination according to this criterion (Fig. 4b). Five trajectory clusters that expanded post all three vaccinations were identified as V160 responsive clusters (Fig. 4c, d). The relative clonal fractions of those five responsive clusters were low (<0.5) or undetected at baseline, suggesting that these clones could be de novo induced by V160 vaccination. Trajectory clusters 30, 41, and 35 showed a V160-induced clonal expansion, peaked and declined sharply at month 7 after the last vaccine dose, and we defined these clusters as exhibiting “transient” expansion in response to V160 (Fig. 4c). Trajectory clusters 37 and 52 also showed a V160-induced clonal expansion but demonstrating very slow contraction post the last dose, and we defined these two trajectory clusters as exhibiting “durable” expansion in response to V160 (Fig. 4d, Supplementary data file 2). The “durable” clonotypes (*n* = 196) collectively constituted about 5% of total TCR sequences at the peak that occurred at month 12, whereas the “transient” clonotypes were more clonally diverse (*n* = 697) but their prevalence peaked at month 7, reaching only 1% of TCR sequences (Fig. 4e). Overall, using longitudinal TCR profiling, we identified two groups of vaccine-responsive T cells with “durable” or “transient” expansion kinetics.

To validate that the “durable” and “transient” clonotypes are indeed HCMV specific, we analyzed CDR3β sequences for recurring motifs. We identified a consensus CDR3β sequence CASSRLAxxxDTQYF that matches public TCR sequences in VDJdb^24^, which have been verified to interact with the HLA-B*08:01-restricted IE-1 epitope (QIKVRVDMV)^25^. Similarly, the consensus sequence CAxxxxGAYNEQFF also matches a public CDR3β sequence against the same IE-1 epitope (QIKVRVDMV) restricted by HLA-B*08:01^26^. We also identified a clonotype with the CDR3β sequence CASSYSGNTEAFF, which has been previously shown to interact with the HLA-B*07:02-restricted pp65 epitope (TPRVTGGGAM)^27,28^. Furthermore, several consensus CDR3β sequences are shared by both “durable” and “transient” clonotypes (Fig. 4f). Taken together, our results show that V160 elicits highly polyclonal T cells.

### V160 induces durable cytotoxic CD8 T cell response

After the vaccine-reactive T cell clonotypes were identified, we sought to better understand these T cells. We annotated the T cells in the scRNA-seq/scTCR-seq data with vaccine-responsive status that were determined from longitudinal TCR profiling by using the CDR3β nucleotide sequences as natural barcodes. By integrating our datasets this way, we can now compare the “durable” vs. “transient” T cells in the single-cell expression data. About 6.45% (45 out of 697) “transient” clones and 48.98% (96 out of 196) “durable” clones matched to scRNA-seq/scTCR-seq data, in which the percentage of responsive CD8 T cells is predominantly higher than responsive CD4 T cells (Fig. 4g). Additionally, V160 responsive CD8 T cells were mostly enriched for “durable” clones, whereas V160 responsive CD4 T cells were mostly enriched for “transient” clones (Fig. 4h).

To validate the results from scRNA-seq and determine the durability of virus-specific T cell responses induced by V160, we did a side-by-side ICS flow cytometry experiment to compare the percentages of IE-1 and pp65 responding CD4 and CD8 T cells in month 9 and month 18 PBMCs from the same subject, with 5 subjects from the 2-dose group and 5 subjects from the 3-dose group. Individual responses were shown in Supplementary Fig. 6. A predominantly higher percentage of antigen-specific CD8 T cells were detected than CD4 T cells in both month 9 and month 18 PBMCs, which is consistent with our scRNA-seq analysis. Interestingly, the average percentages of IE-1 and pp65 responding CD4 and CD8 T cells in the 2-dose group remained stable or showed an upward trend from months 9 to 18. The average percentages of IE-1 and pp65 responding CD4 and CD8 T cells in the 3-dose group had an obvious drop at month 18, mainly due to two high responders (S26 and S28), but still remained at a comparable level to the 2-dose group (Fig. 4i, Supplementary Fig. 6). Notably, one subject (S23) in the placebo group showed dramatically increased T cell responses from month 9 to 18. The titers of HCMV antigen-specific IgG in serum samples of this subject also significantly increased from months 9 to 18 (Supplementary Fig. 7), which indicates HCMV infection. The combinatorial analysis demonstrated that the proportions of total IE-1 responding CD8 T cells and pp65 responding CD4 T and CD8 T cells that co-expressed ≥2 effector molecules at month 18 are comparable to those at month 9 (Fig. 4j, Supplementary Fig. 8). Collectively, these results demonstrate that V160-induced cytotoxic T-cell responses are durable from month 9 to month 18 with a preserved polyfunctionality.

### V160-induced CD8 T cells exhibit a dominant effector phenotype

**Fig. 5:**
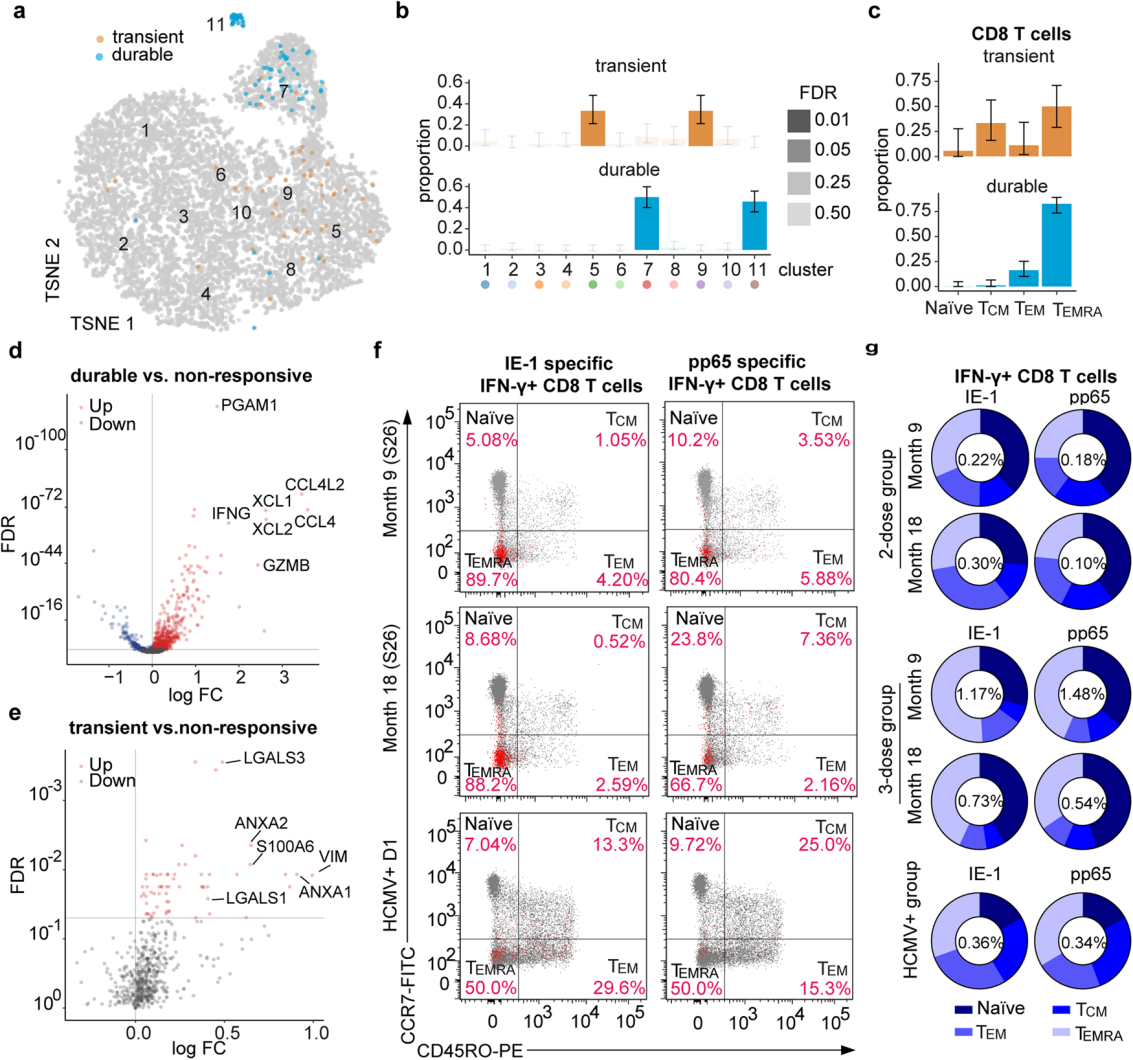
V160-responsive “durable” expanded T cells were dominated by a T_EMRA_ phenotypes. **a** Highlighting of responsive T cells with “transient” or “durable” expansion on the TSNE plot as in Fig. 2a. Statistical significance of the enrichment of each cluster for transient or durable clones was assessed by Fisher’s exact test. **b** Distributions of V160-responsive “transient” vs. “durable” expanded clones across the expression clusters. **c** Distributions of T memory subsets among V160-responsive “transient” or “durable” expanded clones of CD8 T cells, as determined based on single-cell expression profiles. Error bars in **b** and **c** indicate mean ± standard error of the mean. **d** and **e** Volcano plots of scRNA-seq differential gene expression analyses of responsive “durable” T cells versus non-responsive T cells (**d**), or responsive “transient” T cells versus non-responsive T cells (**e**). Each dot summarizes the result for a gene. FDR: false discovery rate. FC fold change. **f** Flow cytometry analysis of memory T cell subsets among IE-1 or pp65 specific CD8 T cells in months 9 and 18 PBMCs of subject S26 and in an HCMV^+^ donor. Antigen-specific T cells identified by IFN-γ expression are shown in red and overlaid with total CD8 T cells shown in gray. **g** Group average distributions of memory T-cell subsets among IE-1 or pp65 specific CD8 T cells in month 9 or 18 PBMCs from 2-dose (*n* = 5), 3-dose (*n* = 5), or HCMV^+^ (*n* = 7) groups. Group average percentage of IFN-γ^+^ CD8 T cells was shown in the center of the pie chart. Individual-level data of memory T cell subset distributions among IFN-γ^+^ CD8 T cells, IL-2^+^ CD8 T cells, TNF-α^+^ CD8 T cells, and CD107a^+^ CD8 T cells are shown in Supplementary Fig. 9.

To better understand the vaccine-responsive “durable” and “transient” clones, we identified the location of these cells in the T-distributed stochastic neighborhood embedding (TSNE) transcriptomic map (Fig. 5a). We found that the “durable” clones are predominantly found in effector cytotoxic T cell clusters 7 and 11, whereas the “transient” clones are predominantly found in the CD4 T cells clusters 5 and 9 (Fig. 5b). Based on single-cell transcriptomic profiles, these vaccine-responsive CD8 T cells were predominantly effector memory T cells of the T_EMRA_ subset, especially for the “durable” clones (Fig. 5c). Furthermore, differential expression analysis of “durable” vs. non-responsive T cells revealed that “durable” clones greatly upregulate many effector-function related chemokines and cytokines such as CCL4, XCL1, XCL2, CCL4L2, and IFNG, as well as cytotoxic protein GZMB (Fig. 5d, Supplementary data file 3). In addition, the “durable” clones also upregulate transcription factors T-bet (TBX21) and eomesodermin (EOMES) and the fractalkine-binding chemokine receptor CX3CR1, which are also expressed by CD8 effector memory T cells induced by HCMV infection in humans^29^. Conversely, the “transient” vs. non-responsive T cells revealed that the “transient” clones upregulate modulators such as Annexin A1 (ANXA1) and Annexin A2 (ANXA2), as well as adhesion molecules such as Vimentin (VIM), LGALS1 (Galectin 1) and LGALS3 (Galectin 3) (Fig. 5e, Supplementary data file 4).

We then validated the effector phenotype of the vaccine-responsive cytotoxic T cells using a flow cytometry assay. By including two additional cell surface markers (CCR7 and CD45RO) into the ICS flow cytometry assay^30^, we were able to differentiate the antigen-specific CD8 T cells into four subsets, the CD45RO^−^CCR7^+^ naïve T cells (naïve), CD45RO^+^CCR7^+^ central memory T cells (T_CM_), CD45RO^+^CCR7^−^ effector memory T cells (T_EM_) and the CD45RO^−^CCR7^−^ subset which corresponds to the T_EMRA_ cells (Supplementary Fig. 1). We analyzed the distributions of four subsets among IE-1 or pp65 specific IFN-γ^+^, CD107A^+^, IL-2^+^, and TNF-α^+^ CD8 T cells in month 9 and month 18 PBMCs from the same subjects and compared to those in HCMV^+^ donors. For S26 in the 3-dose group, T_EMRA_ phenotype accounts for 89.7% of the 3.4% IE-1 specific IFN-γ^+^CD8 T cells at month 9 and 88.2% of the 2.6% IE-1 specific IFN-γ^+^CD8 T cells at month 18; T_EMRA_ phenotype accounts for 80.4% of the 1.5% pp65 specific IFN-γ^+^CD8 T cells at month 9 and 66.7% of the 0.8% pp65 specific IFN-γ^+^CD8 T cells at month 18 (Fig. 5f). The dominance of T_EMRA_ phenotype among IFN-γ^+^CD8 T cells in S26 is consistent with our single cell analysis data. The average proportions of T_EMRA_ phenotype in both 2-dose and 3-dose groups remain stable from month 9 to month 18, though the 2-dose group has lower proportions of T_EMRA_ phenotype and higher proportions of T_EM_ phenotype than the 3-dose group (Fig. 5g). In contrast, the T_EMRA_ and T_EM_ phenotypes together dominate the 1.5% IE-1 IFN-γ^+^CD8 T cells and 0.2% pp65 responding IFN-γ^+^CD8 T cells in HCMV^+^ donors, which are different to those in the 3-dose group but similar to the 2-dose group (Fig. 5f, g, supplementary Fig. 9). Notably, the proportions of total naïve CD8 T cells showed no significant change from month 9 to 18 and are significantly higher compared to HCMV^+^ donors (Supplementary Fig. 10), suggesting minimal perturbation of immune system by V160 vaccination when compared to natural HCMV infection. Together, these results demonstrate that V160-induced CD8 T cells comprise a stable population of T_EMRA_ phenotype that persists for at least one year after the last dose of V160 vaccination.

### Transcriptional signatures of durable CD8 T cells post V160 vaccination

**Fig. 6:**
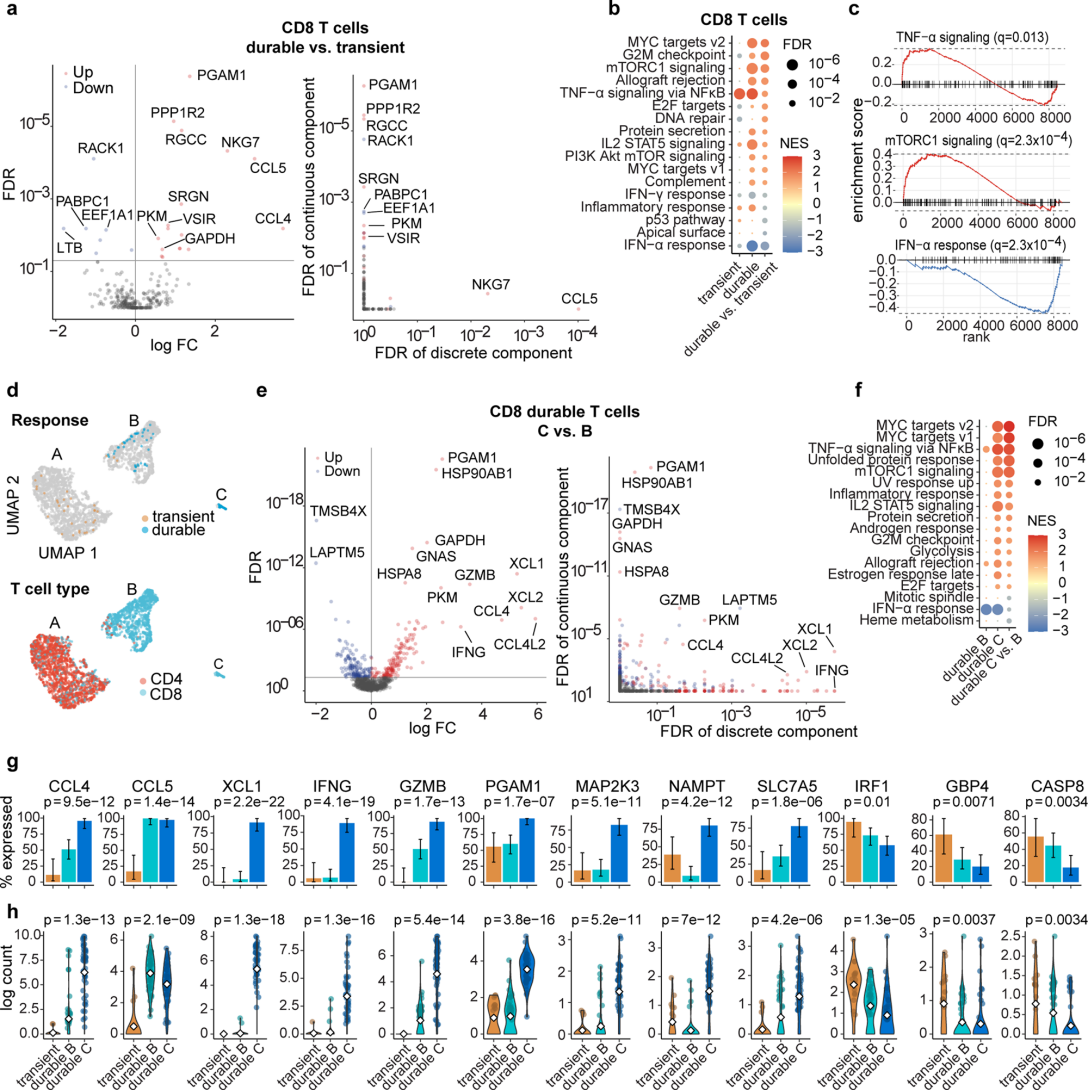
Transcriptional signature of V160-responsive “durable” expanded CD8 T cells. **a** *Left*, Volcano plots of scRNA-seq differential expression analysis of “durable” vs. “transient” expanded CD8 T cells. *Right*, Breakdown of the statistical significance of differential expression into the discrete component (expressed vs. non-expressed) and continuous component (changes in expression level given that the gene is expressed). Each dot summarizes the result for a gene. FDR: false discovery rate. FC: fold change. **b** Summary of gene set enrichment analyses for responsive “transient” vs. non-responsive, responsive “durable” vs. non-responsive, and responsive “durable” vs. “transient” expanded CD8 T cells. NES, normalized enrichment score. **c** Gene set enrichment plots for selected significant pathways in the differential expression of “durable” vs. “transient” expanded CD8 T cells. Dashed lines represent minimum and maximum cumulative enrichment scores. **d** Uniform manifold approximation (UMAP) plots of T cells from expression clusters enriched for “transient” or “durable” expanded V160-responsive clones identified in Fig. 4b. Cells are colored by V160 response type (top) or T cell type (bottom). **e** *Left*, Volcano plots of scRNA-seq differential expression analysis of V160 responsive “durable” CD8 T cells subcluster C vs. B in (**d**). *Right*, Breakdown down the statistical significance of differential expression into the discrete and continuous components. **f** Summary of gene set enrichment analyses for V160 responsive “durable” CD8 T cells subcluster C vs. all other CD8 T cells; subcluster B vs. all other CD8 T cells; or subcluster C vs. B. NES, normalized enrichment score. **g** Percentages of cells expressing selected genes within each V160 responsive T cell cluster. Significance was assessed by Fisher’s exact test. Error bars indicate the mean ± standard error of the mean. **h** Violin plots showing the distributions of log single-cell expression counts in each response group. Each filled dot represents the log expression of a cell expressing the gene. Open diamonds represent mean log expression. Significance was determined by the Kruskal–Wallis test.

We next sought to determine the transcriptional signatures of V160 responsive “durable” CD8 T cells. We compared the single-cell transcriptomes of “durable” versus “transient” CD8 T cells (Fig. 6a). Compared to “transient” CD8 T cells, the “durable” CD8 T cells upregulated the expression of chemokines such as CCL4 and CCL5, which attract immune cells to the site of infection, as well as cytotoxic protein marker NKG7. The “durable” CD8 T cells also expressed higher levels of glycolysis-related enzymes such as PGAM1, PKM, and GAPDH (Fig. 6a, Supplementary data file 5). This preferential use of glycolysis for energy generation is a metabolism feature of effector CD8 T cells^31^. Gene set enrichment analysis showed that the “durable” CD8 T cells activated IL2-STAT5 signaling, which promotes differentiation into terminal effector cytolytic T cells^32,33^, as well as mTORC1 signaling, which is essential for regulation of T-cell glycolytic metabolism^34^. Furthermore, “transient” CD8 T cells activated TNF-α signaling via NF-κB which exerts potent pro-inflammatory functions, while “durable” CD8 T cells showed even higher levels of TNF-α signaling via NF-κB but significantly suppressed interferon-α response (Fig. 6b, c). This could be explained by a cross-regulating relationship between TNF and type I interferons, where TNF controls type I interferons under steady-state conditions^35,36^.

To characterize the relationships among vaccine-responsive T cells more precisely, we performed a UMAP analysis of vaccine-responsive T cells along with T cells that belonged to the same previously determined single-cell expression clusters. This analysis revealed that the V160 responsive “durable” T cells consisted of two subpopulations that we denote as subcluster C and B (Fig. 6d), which correspond to original clusters 11 and 7. We then compared the transcriptional profiles of “durable” CD8 T cells from subcluster C versus B (Fig. 6e, Supplementary data file 6). Subcluster C cells had a dramatic increase in expression of chemokines and cytokines found in activated CD8 T cells, such as XCL1, XCL2, CCL4L2, CCL4, and IFNG, whereas these genes were not expressed in subcluster B (Fig. 6e, g). Consistently, subcluster C also upregulated the expression of glycolysis-related proteins, including PGAM1, PKM, and GAPDH, as well as cytotoxic marker GZMB (Fig. 6e). We speculate that these differences between subclusters C and B should represent V160 responsive cells with or without in vitro stimulation, as IE-1 and pp65 only contain a fraction of potential V160 T-cell epitopes. At the pathway level, “durable” CD8 T cells from subclusters B and C both activated TNF-α signaling via NF-κB and suppressed interferon-α response, whereas only subcluster C cells showed strong activations of many additional pathways such as mTORC1 signaling and IL2-STAT5 signaling (Fig. 6f), suggesting that TNF-α signaling via NF-κB may play a role in the maintenance of “durable” CD8 T cells induced by V160. Overall, “durable” CD8 T cells from subcluster C appeared to show the highest transcript expression of many inflammatory genes, whereas “durable” CD8 T cells from subcluster B showed intermediate expression levels, and “transient” CD8 T cells showed the lowest levels (Fig. 6g, h). Taken together, our results demonstrate that the “transient” and “durable” CD8 T cells induced by V160 vaccination activated distinctive transcriptional programs.

## Discussion

A recent report on a V160 phase 2b clinical trial indicates that V160-induced neutralizing antibody titer is one of the potential correlates of protection (NT50 > 3500) against HCMV infection^9^. Vaccine-induced T-cell responses were measured in about 10% (58–69 subjects) of all participants in each group. Unfortunately, these data were insufficient to identify a relationship between cellular immunity and protection^9^. In this study, we focused on characterizing V160-induced T-cell responses against the de novo synthesized immediate early protein IE-1 and the most abundant viral protein pp65^37,38^. The magnitude of IE-1 and pp65 specific T cells were comparable to those in HCMV^+^ donors and showed a similar polyfunctionality profile upon antigen stimulation in vitro. V160-induced IE-1 and pp65 specific CD8 T cells in the blood showed a potential to be sustainable beyond one year post the last vaccination. Notably, V160-induced IE-1 and pp65 T-cell responses were heterogeneous in both the phase 1 and phase 2b clinical trials^9,23^, which may be related to differences in individual human leukocyte antigen (HLA) backgrounds. Similarly, the strength of IE-1 and pp65 T-cell responses among HCMV^+^ donors are also quite variable^38^. HCMV early and late genes won’t be expressed upon V160 infection due to the absence of IE1/2 and pUL51 proteins. We speculate that T-cell responses directed against the early and late genes should be very low or undetectable unless they exist at high abundance in virions (such as pp65). A comprehensive characterization of V160-induced T-cell responses against the whole HCMV proteome is needed to confirm this hypothesis.

The V160 phase 2b clinical results revealed a 42.4% protective efficacy in the 3-dose V160 group and a −32% protective efficacy in the 2-dose V160 group^8^, suggesting that a low or medium-level immune response by V160 could have an opposite effect. To boost the immune response of V160 for enhanced protection, stronger adjuvants or delivery vehicles, such as Advax and lipid nanoparticle (LNP), could be considered in future clinical trials^39^. The gB/MF59 vaccine mainly induced non-neutralizing antibodies with effector functions for protection against HCMV infection^40^, while the Triplex vaccine induced high-level IE-1/2 and pp65 specific effector memory T cells that are responsible for viremia control in hematopoietic stem cell transplant recipients^12^. Further, a combination of pp65-specific polyfunctional T-cell subsets has been identified to predict protection from subsequent HCMV viremia among HCMV-seropositive lung transplantation recipients, which are protective CD107a^−^IFN-γ^+^IL-2^+^TNF-α^+^ CD4 T-cells and CD107a^−^IFN-γ^+^IL-2^+^TNF-α^+^ CD8 T-cells and the detrimental CD107a^+^IFN-γ^+^IL-2^−^TNF-α^−^ CD8 T-cells^41^. Interestingly, the pp65-specific T cells induced by V160 avoided the detrimental CD8 T subset and were enriched with the protective CD4 T subset. However, it should be noted that these indirect surrogate markers of T-cell activation in response to peptide stimulation may not necessarily directly correlate with antiviral activity and the control of HCMV in vivo, as has been demonstrated by the recent publication of Wills’s group^42^. Future studies are needed to confirm the real antiviral activity of V160-induced T cells.

We identified various V160-induced T-cell clones with distinctive expansion kinetics, together with their unique transcriptomic features in a representative potent T-cell responder S26. Here we observed two potent T-cell responders (S26 and S28) among five 3-dose V160 subjects. Interestingly, a previous phase 1 study revealed three potent T-cell responders among ten 3-dose V160 subjects^23^, suggesting that potent T-cell responders are not rare. Such potent T-cell responses are likely related to specific HLA backgrounds, which should be determined in future studies. Conventional methods to identify antigen-reactive T cells require staining with highly individualized peptide-human leukocyte antigen multimers^43^ or staining with T cell activation markers after in vitro antigen stimulation^44^. These methods are efficient in identifying reactive T cells to a small number of antigens or epitopes, but they usually do not provide a complete picture of T cell responses. We identified V160-responsive total T cell clones in one subject through longitudinal analysis of blood TCR expansion kinetics and found two distinctive “transient” or “durable” expansion kinetics exhibited by the V160-responsive T cell clones. Despite the lower diversity of the “durable” clones, their total frequencies were much higher than the “transient” clones at all time points post-vaccination. Consistent with flow cytometry analysis, the “durable” clones were enriched for CD8 T cells with a predominant T_EMRA_ phenotype, and the “transient” clones were mostly enriched for CD4 T cells. Differentially expressed genes of “durable” T cells induced by V160 are consistent with the phenotypes, effector functions, metabolism, and feature transcription factors of the long-lived CD8 effector T cells found in HCMV^+^ individuals^19,29,31^. After a step-wise comparison, we identified that activation of the TNF-α signaling via NF-κB pathway may contribute to the maintenance of “durable” CD8 effector T cells induced by V160. Such knowledge helps us to understand the molecular pathways involved in the activation, differentiation, and maintenance of HCMV-specific durable CD8 T cells. Longitudinal scRNA-seq data at additional time points other than Month 18 will be needed to find out the mechanisms behind the rapid decline of the “transient” clones and the development of the “durable” clones in future studies.

T-cell immunity to cytomegalovirus infection is characterized by the accumulation of a large population of circulating memory CD8 T cells with an effector phenotype. The phenomenon has been termed “memory inflation” on the basis of longitudinal studies in mouse models^45^. Similarly, we found that the majority of V160-induced CD8 T cells at both months 9 and 18 did not express CCR7, a chemokine receptor that directs the migration of lymphocytes to lymph nodes^46^. Due to the propensity of HCMV to elicit long-term T cell immunity of unparalleled strength, there has been extensive interest in developing HCMV-vectored vaccines for the induction of persistent T cell immunity against other targets, including viruses (influenza, Ebola, and HIV)^47–49^, mycobacterium tuberculosis^50^, as well as cancers^51–53^. There is also interest in redirecting the widely existent HCMV-specific CD8 T cells in humans for reducing HCMV-related diseases in transplant recipients as well as for cancer immunotherapy^22,54–56^. Murine cytomegalovirus (MCMV) is among the best-studied models for the mechanisms behind potent T-cell immunity of cytomegalovirus. Hill et. al found that single-cycle MCMV, which cannot spread from initially infected cells due to the lack of gL, can still generate viral antigens to drive CD8 T cell memory inflation in mice only if the virus was administered systemically^57^. Using recombinant MCMV carrying foreign T-cell epitopes, Cicin-Sain et al. showed that constitutive proteasome processing of antigenic epitopes in latently infected cells is required for the induction of robust inflationary T-cell responses in mice^53^. Here we showed that V160, a replication-defective virus, can induce IE-1 and pp65-specific CD8 T cells that are very similar to those observed in HCMV^+^ individuals in terms of magnitude, phenotype, and functionality. We speculate that the potent T cell immunity induced by V160 could be attributed to its following features: (1) ddFKBP-mediated constitutive proteasome degradation of viral IE-1/2 and pUL51 for epitope presentation, (2) the abortive life cycle of V160 in normal cells due to its replication defective feature, and (3) the broad cell tropism enabled by the gH/gL/pUL128/130/131 complex. Critically, our study showed that HCMV replication may not be required for the induction of sustained effector memory T cells in humans, which thus provides important implications for the design of vaccines based on HCMV vectors for robust T cell immunity.

Our study on T-cell responses to V160 after vaccination in humans should have broad impacts on the research of HCMV immunology, modified HCMV-vectored vaccines, and other rationally designed vaccines that rely on robust effector T-cell immunity. Meanwhile, we recognize that there are several limitations to this study. The most obvious one is the lack of correlative studies between T-cell immune responses and vaccine efficacy. Given the expected genetic diversity among human subjects and the complexity of T-cell assay readouts, our data lack sufficient power for clear differentiation of dosing regimens due to the limited sample size. Due to the scarcity of PBMC samples, we only analyzed the IE-1 and pp65 specific T cell responses by flow cytometry, despite the fact that natural T cell immunity against HCMV infection can target a much broader range (~151 ORFs) of antigens^18^. Extended follow-ups and more samples from the V160 subjects would be needed for future studies to determine the long-term impacts of V160 vaccination on the immune system in comparison to natural HCMV infection and to understand the mechanisms behind the rapid decline of the “transient” clones and development of the “durable” clones.

## Methods

### Study subjects and samples

This study aimed to evaluate T-cell responses elicited by V160. It is a sub-study of a phase 2b clinical trial (V160-002). The V160-002 trial is a double-blind, randomized, placebo-controlled phase 2b, multi-center study to evaluate the safety, tolerability, efficacy and immunogenicity of a 2-dose and a 3-dose regimen of HCMV vaccine (V160) in healthy seronegative women at 16–35 years of age (https://clinicaltrials.gov/ct2/show/NCT03486834). The trial was performed in conformance with the standards of Good Clinical Practice. The protocol was reviewed and approved by the Western Institutional Review Board, Inc., and the Institutional Review Board of the University of Texas Medical Branch. All subjects provided written informed consent before participation. Human samples were collected from study subjects with informed consent at indicated study time points as described below. Subjects received blinded V160 on Day 1 (D1), Month 2 (M2), and M6 (3-dose regimen), V160 on D1 and M6 and placebo at M2 (2-dose regimen), or placebo on D1, M2, and M6. V160 (100 units) or saline solution containing Merck aluminum phosphate adjuvant (MAPA) was administered as a 0.5 ml intramuscular (IM) injection. Twenty-one subjects enrolled at the UTMB site participated in the T-cells study. A small amount of blood (2.5 ml) was collected for isolation of serum and whole blood RNA samples at D1, M2, M6, M7, M9, M12, M18, and M24, and a large amount of blood (100 ml) was collected at M9 and M18 for isolation of PBMCs. In the end, 8 subjects in 2-dose V160 group, 6 subjects in 3-dose V160 group and 4 subjects in placebo group made it through month 7; 7 subjects in 2-dose V160 group, 5 subjects in 3-dose V160 group and 4 subjects in placebo group made it through month 9 for collection of PBMCs; 5 subjects in 2-dose V160 group, 5 subjects in 3-dose V160 group and 3 subjects in placebo group made it through month 18 for collection of PBMCs. Serum samples and blood collected in PAXgene tubes for RNA isolation were stocked at −80 °C until usage. Deidentified buffy coat samples from seven age- and sex-matched HCMV seropositive and four HCMV seronegative healthy donors were purchased from Gulf Coast Regional Blood Center. PBMCs were isolated from fresh blood or buffy coat using ACCUSPINTM system-Histopaque (Cat#A7054, Sigma) according to the manufacturer’s instructions. All PBMCs were frozen in a cell freezing medium (Cat. Log 302-14681, BAMBANKER) at 1 × 10^7^ cells/vial and stocked in liquid nitrogen until usage.

### Antigen peptides

A pool of 120 peptides (15 mers with 11 amino acids overlap) spanning the HCMV IE-1 and a pool of 138 peptides (15 mers with 11 amino acids overlap) spanning the HCMV pp65 were purchased from JPT Peptide Technology (Product code: PM-IE-1 and PM-pp65-2). The peptide pools were dissolved in DMSO at a concentration of 0.4 mg/ml for each peptide and stocked at −80 °C freezer until usage.

### Flow cytometry and data analysis

Frozen PBMCs were recovered in R10 medium (RPMI1640 supplemented with 10% inactivated FBS, 2-Mercaptoethanol, HEPES Buffer, l-glutamine, sodium pyruvate, and penicillin–streptomycin) and rested overnight in 37 °C incubator with 5% CO_2_. The next day, live PBMCs were counted by trypan blue exclusion on a hemocytometer. Then, 1 × 10^6^ PBMCs (per sample) in R10 medium were stimulated in a round-bottomed 96-well plate with DMSO, staphylococcal enterotoxin B (SEB) (Cat: S4881, Sigma), IE-1 peptide pool, or pp65 peptide pool in presence of 1 μg/ml anti-CD28/CD49d, 5 μg/ml monensin (Cat: M5273, Sigma), 5 μg/ml brefeldin A (BFA) (Cat: B7651, Sigma) and 1/50 diluted anti-CD107a-APC H7 (Cat: 561343, BD) in a final volume of 200 μl for 6 h in an incubator with 5% CO_2_ at 37 °C. The anti-CD28 (Cat: 340975, BD Biosciences) and anti-CD49d (Cat: 340976, BD Biosciences) were mixed at a ratio of 1:1 and served as a co-stimulator. The addition of BFA and monensin prevents cytokines released from cells during stimulation. The final concentration of each peptide in IE-1 and pp65 peptide pools was 2 μg/ml. For PBMCs from each subject, one sample stimulated with an equal amount of DMSO solvent (1 μl per test) and one sample stimulated with 2 μg/ml SEB at the same conditions as that of peptide pools served as negative and positive controls, respectively. After stimulation, the cells were firstly stained for live/dead using the Violet Live/Dead kit (Cat: L34955, Invitrogen) at room temperature (RT) for 15 min and followed by a washing step. Then, the cells were stained by an antibody cocktail (150 μl per test) which contains 1/50 diluted anti-CD4-AF700 (Cat: 566318, BD), 1/300 diluted anti-CD8-BUV395 (Cat: 563798, BD), 1/30 diluted anti-CD45RO-PE (Cat: 12-0457-42, Invitrogen), and anti-CD197(CCR7)-FITC (Cat: 353216, BioLegend) for 45 min at 4 °C. After a washing step, the cells were further treated with Cytofix/Cytoperm (Cat: 554722, BD) and then stained by an antibody cocktail (150 μl per test) which contains 1/75 diluted anti-CD3-ECD (Cat: IM2705U, Beckman Coulter) and 1/30 diluted anti-TNF-α-PE-Cy7 (Cat: 557647, BD), anti-IFN-γ-V500 (Cat: 561980, BD) and anti-IL-2-APC (Cat: 341116, BD) at room temperature for 30 min. After a washing step, the stained cells were suspended in a stabilizing fixative buffer (Cat: 338036, BD). Data were acquired using BD Aria II SORP cytometer (250,000 cells were recorded per sample).

Flow cytometry data were analyzed with FlowJo software (V9.7.6). CD4 T and CD8 T cells were identified by sequential gating (Supplementary Fig. 1a). Briefly, lymphocytes were identified by gating of SSC-A and FSC-A. Singlets were gated from lymphocytes using FSC-W and FSC-H and followed by gating for CD3^+^ live cells. The CD4 T cells and CD8 T cells were identified from live CD3^+^ T cells through the gating of CD4 and CD8. The differentiation status of total (or antigen-specific) CD4 T and CD8 T cells was determined by gating on CCR7 and CD45RO (Supplementary Fig 1b, c). Antigen-specific CD4 T cells and CD8 T cells were identified by gating on four effector molecules (CD107a, IFN-γ, IL-2, and TNF-α) separately (Supplementary Fig 1d, e) and shown as background (DMSO) subtracted data (Supplementary data file 1). Positive cutoffs of antigen-specific T cells for four effectors were calculated from median plus two-fold standard error of the mean of all the negative controls (DMSO stimulation, 27 individuals) in this study (Supplementary Fig. 2), which were 0.056% CD107a^+^ CD4 T, 0.081% IFN-γ^+^ CD4 T, 0.11% IL-2^+^ CD4 T, 0.55% TNF-α^+^ CD4 T, 0.19% CD107a^+^ CD8 T, 0.25% IFN-γ^+^ CD8 T, 0.084% IL-2^+^ CD8 T, and 0.30% TNF-α^+^ CD8 T, respectively. A positive response is defined as >corresponding specificity cutoff. A positive responder is defined as an individual with at least one positive response. For polyfunctional analysis, a Boolean combination gate was applied on four subpopulations of CD4 T (or CD8 T) cells that were gated on CD107A, IFN-γ, IL-2, and TNF-α, respectively. The percentages of CD4 T (or CD8 T) cells that expressed 16 combinations of the four effector molecules were obtained for data analysis using SPICE 6.0. Data were background subtracted and visualized using GraphPad Prism 8.

### Enrichment of CD3^+^ T cells for single-cell sequencing

PBMCs were thawed and rested overnight in R10 medium. Cells (5–6 × 10^6^/ml) were stimulated with IE-1 and pp65 peptide pools (2 μg/ml/peptide) in the presence of 1.0 μg/ml anti-CD28/CD49d antibodies for 5 h at 37 °C. Cells were washed with PBS and followed by staining with LIVE/DEAD™ fixable violet stain (Invitrogen). Then the cells were washed and stained with 1/75 diluted anti-human CD3 ECD (Cat: IM2705U, Beckman Coulter). Immediately after staining, single cells gated on live CD3^+^ lymphocytes were sorted on a BD Aria II SORP cytometer. The cells were used for single-cell sequencing within 2 h after sorting.

### Single-cell RNA library generation and sequencing

Flow-sorted cells (500 cells/µl) in complete RPMI medium plus 10% FBS were applied to the Chromium single-cell controller for generation of single-cell droplets and barcoding using the Chromium Single-cell 5’ reagent version 2 kit according to the manufacturer’s protocol. A total of 10,000 cells were targeted. Reverse transcription, RT-cleanup, and cDNA amplification were performed and followed by the construction of 5′ gene or enriched V(D)J libraries using the V(D)J Human T Cell Enrichment Kit, Library Construction Kit, and i7 Multiplex Kit. Quality controls and concentrations of cDNA and libraries were determined using Agilent Bioanalyzer High sensitivity chip on Agilent 2100 Bioanalyzer and Qubit Fluorometer. Libraries were sequenced on an Illumina NovaSeq 600 platform with sequencing parameters of 26-10-10-90. The scRNAseq 5’ gene libraries were sequenced to a read depth of >70,000 reads per cell. The TCR V(D)J enriched libraries were sequenced to a read depth of ~ 20,000 reads per cell.

#### Single-cell RNA sequencing data analysis

Read preprocessing, alignment, quantification, empty droplet removal, and sample aggregation for the 5’ expression data were performed using 10x Genomics Cell Ranger pipelines (v6.0.1). The count’s data were then imported into R using DropletUtils^58^. Quality control and additional preprocessing were conducted using Scater and scuttle^59^; barcodes with library size factor ≤0.2, number of detected features ≤600, and mitochondria gene expression ≥8 were removed. Principle component analysis, uniform manifold approximation and projection^60^, t-stochastic neighbor embedding^61^, as well as cell clustering were performed using scran^62^. After modeling mean-variance in the log-expression profiles for each gene, the expression data was denoised by removing principal components that likely correspond to technical noise^62^. As part of quality control, an initial clustering of the cells was conducted based on short random walks on a nearest-neighbor graph using bluster in order to identify small cell clusters^62^. Because the input cells to scRNA-seq were CD3^+^ T cells, non-T cells were identified and removed by excluding cell clusters with high expressions of markers for B cells, dendritic cells, and monocytes, which likely arose from doublets and/or cell-debris complexes^63^. A subsequent cell clustering of T cells was performed using the Louvain method^64^ in order to identify subpopulations of T cells based on single-cell expression patterns. Differential scRNA-seq expression analyses were performed using the hurdle model from MAST^65^. Gene set enrichment analyses were conducted by testing for average differences in expressions of genes in the gene set and using bootstrapping to estimate the between-gene covariance, as implemented in MAST^65^. Additionally, the gene-level signed Wald statistics were calculated using coefficient estimates and standard errors from MAST differential gene expression analyses, and these statistics were used to perform additional gene set enrichment analyses using the enrichment score-based method implemented in fgsea^66^. Hallmark gene sets were retrieved from the Molecular Signatures Database^67^.

Single-cell TCR sequence analysis, aggregation, and clonotype assignment were performed using 10× Genomics Cell Ranger pipelines. TCR clonotypes were annotated in the scRNA-seq data by matching 10× barcode sequences identified here with those in the single-cell 5’ expression data.

### Blood TCRβ library generation and sequencing

Eight PAXgene whole blood RNA samples collected at D1 (M0), M2, M6, M7, M9, M12, M18 and M24 from subject 26 were used for TCRβ repertoire sequencing. Total blood RNAs were isolated using a PAXgene blood RNA kit (Cat. No. 160048338, PreAnalytiX) following the manufacturer’s instructions. The quality of RNA samples was determined with an Agilent Bioanalyzer and then quantified using Qubit 2.0. For each time point, 500 ng RNA (RIN > 7.1) was used to generate TCRβ only libraries using Takara SMARTer® Human TCR α/β Profiling Kit v2 (Cat. No. 634481) following manufacturer’s instructions. The libraries were purified using Takara NucleoMag NGS Clean-up and Size Select beads. All eight libraries were quantified for the concentration of adapter-ligated fragments using qPCR and then pooled equimolarly. For sequencing, 0.9 pM of equimolarly pooled library is loaded onto a NextSeq 500 Mid Output v2.5 flowcell (Illumina p/n 20024904) and amplified by bridge amplification using the Illumina NextSeq 500 sequencing instrument. PhiX Control v3 adapter-ligated library (Illumina p/n FC-110-3001) is spiked-in at 20% by weight to ensure balanced diversity and to monitor clustering and sequencing performance. A paired-end 150-cycle run was used to sequence the flowcell on a NextSeq 500 Sequencing System. An average of 12.8 million read pairs was sequenced per sample.

#### Longitudinal TCRβ sequence data analysis

The sequence data was processed using the Cogent NGS Immune Profiler software. A total of 2,318,537 TCR CDR3β clones were detected, and 1,936,450 were unique clones. After unique TCR CDR3β sequences were filtered for those that are supported by ≥2 reads and detected in ≥ 3 samples, 33,074 clones were obtained. The log relative clonal fractions ${r}_{{jt}}$ of CDR3β sequence $j$ at the time point $t$ were calculated using two formulas as shown below:

$${r}_{{jt}}=\log \left(\frac{{f}_{{jt}}}{\frac{1}{T}\mathop{\sum }\limits_{t}^{T}{f}_{{jt}}}\right)$$

$${f}_{{jt}}=\frac{\alpha +{c}_{{jt}}}{\alpha J+{l}_{t}}$$

where $T$ is the number of time-point samples which is eight here, ${f}_{{jt}}$ is clonal fraction, $J$ is the number of unique CDR3β sequences, and $\alpha$ is a pseudo-count smoothing hyperparameter. Additionally, ${c}_{{jt}}$ is the number of reads mapping to clone $j$ at time point $t$ (i.e. read count) after filtering, and the library size factor ${l}_{t}=\mathop{\sum }\nolimits_{j}^{J}{c}_{{jt}}^{0}$ is calculated based on read counts ${c}_{{jt}}^{0}$ before filtering of the CDR3β sequences. In this way, the log relative clonal fraction vector ${{\boldsymbol{r}}}_{j}={({r}_{{jt}})}_{t=1}^{T}$ represents the temporal trajectory of how T cell clone $j$ changes in abundance over time. The T cell clones represented by the CDR3β sequences were then clustered based on ${{\boldsymbol{r}}}_{j}$ using *K*-means clustering (*K* = 100) in order to identify trajectory clusters of T cell clones. The trajectory clusters were then plotted on a linear scale and manually inspected to identify vaccine-responsive clones that started with low or undetected baseline clonal fractions and exhibited consistent increases after each vaccine dose. Five trajectory clusters met this requirement with baseline relative clonal fractions <0.5. Three clusters (30, 35, 41) whose clonal fractions peaked at month 7 and declined sharply at month 9 were denoted as “transient” expanded clones in response to V160. Two clusters (37, 52) whose clonal fractions remained stable or elevated after the last dose were denoted as “durable” expanded clones in response to V160.

Vaccine-reactive T cells were annotated in the scRNA-seq data by matching CDR3β sequences identified here in the longitudinal TCR sequence analysis with those in the scRNA-seq data. TCR CDR3β sequence clusters and motifs were identified using GIANA^68^ and GLIPH2^69^. About 15.8% that is 141 out of 893 responsive clones matched to scRNA-seq clones by TCRB CDR3 sequence. Among the 141 clones, there are 28 CD4 T cells which are composed of “26” transient clones and 2 “durable” clones; there are 113 CD8 T cells which are composed of 19 “transient” clones and 94 “durable” clones.

#### Virus neutralization assay

Serum samples were heat-inactivated at 56°C for 30 min before the experiment. A virus neutralization assay was performed in a 96-well plate using ARPE-19 cells and HCMV strain AD169rev-GFP. Briefly, 50 µl 2-fold serially diluted serum samples were mixed with equal volumes of diluted AD169rev-GFP that produces about 100 GFP foci in each well and incubated at 37 °C for 30 min before adding to confluent ARPE-19 cells. Mock-infected cells and cells infected with the virus only served as controls. Triplicate wells were determined for each condition. Viral infection was examined at 48 h after infection. A C.T.L. ImmunoSpot analyzer was used to capture whole-well images of GFP expression and quantification of GFP-positive cells. The percentage of viral inhibition by the serum sample was calculated, and the NT50 of each sample was derived by a nonlinear fit of virus inhibition % versus dilution factor using GraphPad Prism 5 software.

#### Endpoint IgG titer by ELISA assay

Costar 96-well high binding plates were coated with 200 ng/well of recombinant gB (32–692 aa), 200 ng/well of soluble pentamer, or 100 ng/well of inactivated virion in PBS overnight at 4 °C, respectively. Then after each of the following steps, the plate was washed three times with PBST buffer (PBS with 0.05% Tween 20). All samples and antibodies were diluted using 1% non-fat milk. The plate was blocked with 200 μl/well of 5% non-fat milk for 1 h at 37 °C, incubated with 100 μl/well of 2-fold serially diluted serum samples at 37 °C for 1 h and detected with 100 μl/well of 1/5000 diluted goat anti-human IgG HRP (Cat: 109-035-088, Jackson lab). After color development with TMB substrate, absorbance at 450 nm was recorded on a Molecular Devices Spectra Max M4 machine. The highest dilution that produces OD450 reading at least 0.1 above background (detection antibody only) was determined as endpoint IgG titer.

#### Statistical analysis

Analyses were performed in R environment (v4.1.2) with R packages from Bioconductor (v3.14) unless indicated. Whenever applicable, *p* values were adjusted for multiple hypothesis testing using the Benjamini–Hochberg method^70^.

### Reporting summary

Further information on research design is available in the Nature Research Reporting Summary linked to this article.

### Supplementary information

Supplementary Data file 1

Supplementary Data file 2

Supplementary Data file 3

Supplementary Data file 4

Supplementary Data file 5

Supplementary Data file 6

REPORTING SUMMARY

Supplementary Figures

## Data Availability

The scRNA-seq and scTCR-seq data, as well as TCR profiling data in this study, have been deposited in the Sequence Read Archive with accession numbers PRJNA832855 and PRJNA832878. All other relevant data supporting the findings of this study are available within the article and its Supplementary data files or from the corresponding authors upon reasonable request.

## Code availability

The code supporting this study is available at the GitHub repository https://github.com/djhshih/analysis-v160.
