# Supplementary material for: Transcriptional signature of durable effector T cells elicited by a replication defective HCMV vaccine
Source: NPJ Vaccines. 2024 Apr 1;9:70. doi: 10.1038/s41541-024-00860-w (PMC10984989; doi:10.1038/s41541-024-00860-w)
Supplement: Supplementary file 7 — REPORTING SUMMARY [file 41541_2024_860_MOESM7_ESM.pdf]

nature portfolio

Corresponding author(s): Professor Zhiqiang An

Last updated by author(s): Feb 5, 2024

Reporting Summary

Nature Portfolio wishes to improve the reproducibility of the work that we publish. This form provides structure for consistency and transparency in reporting. For further information on Nature Portfolio policies, see our Editorial Policies and the Editorial Policy Checklist.

Statistics

For all statistical analyses, confirm that the following items are present in the figure legend, table legend, main text, or Methods section.

n/a	Confirmed	
☐	☒	The exact sample size (*n*) for each experimental group/condition, given as a discrete number and unit of measurement
☐	☒	A statement on whether measurements were taken from distinct samples or whether the same sample was measured repeatedly
☐	☒	The statistical test(s) used AND whether they are one- or two-sided<br>*Only common tests should be described solely by name; describe more complex techniques in the Methods section.*
☒	☐	A description of all covariates tested
☐	☒	A description of any assumptions or corrections, such as tests of normality and adjustment for multiple comparisons
☐	☒	A full description of the statistical parameters including central tendency (e.g. means) or other basic estimates (e.g. regression coefficient) AND variation (e.g. standard deviation) or associated estimates of uncertainty (e.g. confidence intervals)
☐	☒	For null hypothesis testing, the test statistic (e.g. *F*, *t*, *r*) with confidence intervals, effect sizes, degrees of freedom and *P* value noted<br>*Give P values as exact values whenever suitable.*
☒	☐	For Bayesian analysis, information on the choice of priors and Markov chain Monte Carlo settings
☒	☐	For hierarchical and complex designs, identification of the appropriate level for tests and full reporting of outcomes
☒	☐	Estimates of effect sizes (e.g. Cohen's *d*, Pearson's *r*), indicating how they were calculated

Our web collection on statistics for biologists contains articles on many of the points above.

Software and code

Policy information about availability of computer code

Data collection	For scRNA-seq, read preprocessing, alignment, quantification, empty droplet removal, and sample aggregation for the 5' expression data were performed using 10x Genomics Cell Ranger pipelines (v6.0.1). The longitudinal TCR beta sequence data was processed using the Cogent NGS Immune Profiler software. TCR CDR3β sequence clusters and motifs were identified using GIANA and GLIPH2. Please see details in the method section.
Data analysis	Code supporting this study is available at the GitHub repository https://github.com/djhshih/analysis-v160

For manuscripts utilizing custom algorithms or software that are central to the research but not yet described in published literature, software must be made available to editors and reviewers. We strongly encourage code deposition in a community repository (e.g. GitHub). See the Nature Portfolio guidelines for submitting code & software for further information.

Data

Policy information about availability of data

All manuscripts must include a data availability statement. This statement should provide the following information, where applicable:

- Accession codes, unique identifiers, or web links for publicly available datasets
- A description of any restrictions on data availability
- For clinical datasets or third party data, please ensure that the statement adheres to our policy

The scRNA-seq and scTCR-seq data, as well as TCR profiling data, in this study have been deposited in the Sequence Read Archive with accession numbers

PRJNA832855 and PRJNA832878. All other relevant data supporting the findings of this study are available within the article and its Supplementary data files, or from the corresponding authors upon reasonable request.

Research involving human participants, their data, or biological material

Policy information about studies with human participants or human data. See also policy information about sex, gender (identity/presentation), and sexual orientation and race, ethnicity and racism.

Reporting on sex and gender	Not applicable
Reporting on race, ethnicity, or other socially relevant groupings	Not applicable
Population characteristics	Healthy HCMV seronegative women at 16~35 years of age
Recruitment	This study is a sub-study under V160-002 clinical trial. CMV-seronegative (assessed by LIAISON CMV IgG immunoassay [DiaSorin, Saluggia, Italy]), non-pregnant women of childbearing potential, aged 16–35 years, were eligible to participate in the study. Other inclusion criteria included being deemed healthy on the basis of medical history and physical examination and having exposure to children aged 5 years or younger at home or work. Eligible participants agreed to avoid becoming pregnant until 4 weeks after the last dose of V160 or placebo. Key exclusion criteria included: hypersensitivity to a vaccine component, known or suspected impairment of immunological function, recent febrile illness, or previous receipt of a CMV vaccine. Please see other details in the publication https://doi.org/10.1016/S1473-3099(23)00343-2.
Ethics oversight	The protocol was reviewed and approved by the Western Institutional Review Board, Inc., and the Institutional Review Board of the University of Texas Medical Branch.

Note that full information on the approval of the study protocol must also be provided in the manuscript.

Field-specific reporting

Please select the one below that is the best fit for your research. If you are not sure, read the appropriate sections before making your selection.

☒ Life sciences ☐ Behavioural & social sciences ☐ Ecological, evolutionary & environmental sciences

For a reference copy of the document with all sections, see nature.com/documents/nr-reporting-summary-flat.pdf

Life sciences study design

All studies must disclose on these points even when the disclosure is negative.

Sample size	Twenty-one participants enrolled at the UTMB site participated in the T-cells study.8 subjects in 2-dose V160 group, 6 subjects in 3-dose V160 group and 4 subjects in placebo group made it through month 7; 7 subjects in 2-dose V160 group, 5 subjects in 3-dose V160 group and 4 subjects in placebo group made it through month 9 for collection of PBMCs; 5 subjects in 2-dose V160 group, 5 subjects in 3-dose V160 group and 3 subjects in placebo group made it through month 18 for collection of PBMCs.
Data exclusions	No data were excluded from the analysis.
Replication	The flow cytometery, ELISA and neutralization experiments have been performed twice with similar results. ScRNA-seq and blood RNA-seq was done one time.
Randomization	Investigators and site staff enrolled participants using central randomisation via an interactive response technology system in blocks of six for assignment to vaccination groups. Participants were randomly assigned 1:1:1 to one of the three groups.
Blinding	Participants and Investigators were double binded for the V160-002 clinical trial.

Reporting for specific materials, systems and methods

We require information from authors about some types of materials, experimental systems and methods used in many studies. Here, indicate whether each material, system or method listed is relevant to your study. If you are not sure if a list item applies to your research, read the appropriate section before selecting a response.

Materials & experimental systems

n/a	Involved in the study
☐	☒ Antibodies
☐	☒ Eukaryotic cell lines
☒	☐ Palaeontology and archaeology
☒	☐ Animals and other organisms
☒	☐ Clinical data
☒	☐ Dual use research of concern
☒	☐ Plants

Methods

n/a	Involved in the study
☒	☐ ChIP-seq
☐	☒ Flow cytometry
☒	☐ MRI-based neuroimaging

Antibodies

Antibodies used	Anti-CD3-ECD (Cat: IM2705U, BECKMAN COULTER), anti-CD4-AF700 (Cat: 566318, BD), anti-CD8-BUV395 (Cat: 563798, BD), anti-CD45RO-PE (Cat: 12-0457-42, Invitrogen), anti-TNF-α-PE-Cy7 (Cat: 557647, BD), anti-IFN-γ-V500 (Cat: 561980, BD), anti-CD107a-APC H7 (Cat: 561343, BD), and anti-IL-2-APC (Cat: 341116, BD), and anti-CD197(CCR7)-FITC (Cat: 353216, BioLegend)
Validation	All antibodies used in this study were commercial antibodies that have been well validated. Please see details on the website.

Eukaryotic cell lines

Policy information about cell lines and Sex and Gender in Research

Cell line source(s)	ARPE-19 cells, ATCC (CRL-230)
Authentication	The cell line was not authenticated.
Mycoplasma contamination	The cell line was not tested for mycoplasma contamination
Commonly misidentified lines (See ICLAC register)	None

Plants

Seed stocks	Not applicable
Novel plant genotypes	Not applicable
Authentication	Not applicable

Flow Cytometry

Plots

Confirm that:

☒ The axis labels state the marker and fluorochrome used (e.g. CD4-FITC).

☒ The axis scales are clearly visible. Include numbers along axes only for bottom left plot of group (a 'group' is an analysis of identical markers).

☒ All plots are contour plots with outliers or pseudocolor plots.

☒ A numerical value for number of cells or percentage (with statistics) is provided.

Methodology

Sample preparation	PBMCs were were isolated from fresh blood or buffy coat using ACCUSPINTM system-Histopaque (Cat#A7054, Sigma) according to manufacturer's instructions. All PBMCs were frozen in cell freezing medium (Cat. Log 302-14681, BAMBANKER) at 1×10^7 cells/vial and stocked in liquid nitrogen until usage.
Instrument	BD Aria II SORP cytometer

Software	FlowJo software (V9.7.6)
Cell population abundance	The abundance of CD3+ T cells post sorting was >99%.
Gating strategy	CD4 T and CD8 T cells were identified by sequential gating as shown in Supplementary Fig 1a. Lymphocytes were identified by gating of SSC-A and FSC-A. Singlets were gated from lymphocyte using FSC-W and FSC-H and followed by gating for CD3+ live cells. The CD4 T cells and CD8 T cells were identified from live CD3+ T cells through gating of CD4 and CD8. Then, the CD4 T cells and CD8 T cells were analyzed for expression of four effector molecules (CD107a, IFN-γ, IL-2, and TNF-α) separately as shown in Supplementary Fig 1b-c. Effector phenotypes of CD4 T and CD8 T cells were determined by gating on expression of CCR7 and CD45RO as shown in Supplementary Fig 1d-e. For polyfunctional analysis, a Boolean combination gates was applied on four subpopulations of CD4 T (or CD8 T) cells that were gated on CD107A, IFN-γ, IL-2 and TNF-α, respectively.

☒ Tick this box to confirm that a figure exemplifying the gating strategy is provided in the Supplementary Information.
