# Supplementary material for: Transcriptional signature of durable effector T cells elicited by a replication defective HCMV vaccine
Source: NPJ Vaccines. 2024 Apr 1;9:70. doi: 10.1038/s41541-024-00860-w (PMC10984989; doi:10.1038/s41541-024-00860-w)
Supplement: Supplementary file 8 — Supplementary Figures [file 41541_2024_860_MOESM8_ESM.pdf]

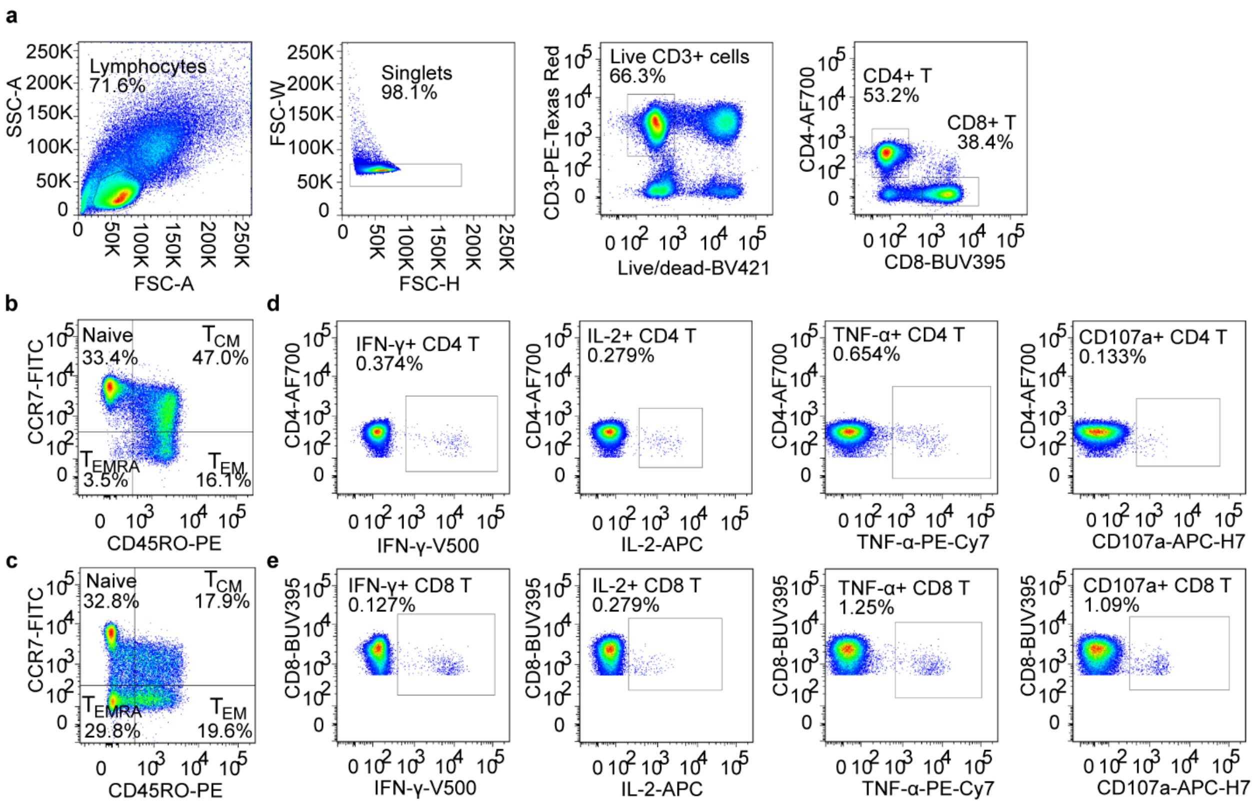

Supplementary Fig 1. Representative gating strategy of flow cytometry analysis. **a**, Sequential gating for CD4 T and CD8 T cells. The lymphocytes populations were identified from PBMCs based on gating of SSC-A and FSC-A. Singlets were gated from lymphocyte using FSC-W and FSC-H and followed by gating on CD3 and live/dead staining. The CD4 T cells and CD8 T cells were identified from live $CD3^+$ T cells through gating of CD4 and CD8. **b-c**, Differentiation status of (**b**) CD4 T and (**c**) CD8 T cells were identified by gating on CCR7 and CD45RO. **d-e**, Antigen specific (**d**) CD4 T and (**e**) CD8 T cells were identified by gating on four effector molecules (CD107a, IFN-γ, IL-2, and TNF-α) separately. The data shown were from pp65 stimulated PBMCs of a $HCMV^+$ Donor.

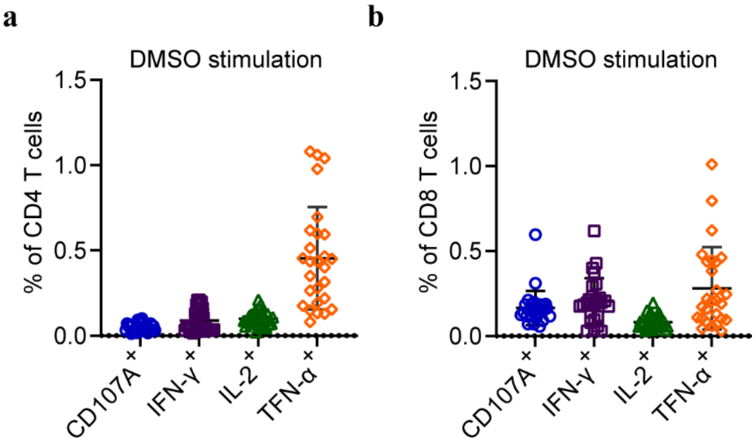

Supplementary Fig 2. Background T-cell responses. Percentages of (**a**) CD4 T and (**b**) CD8 T cells that were positive for the four effector molecules upon DMSO stimulation, respectively. Data were from a total of 27 individuals, including seven 2-dose subjects, five 3-dose subjects, four placebo subjects, seven HCMV$^+$ donors, and four HCMV$^-$ donors. Each symbol represents one individual. Bars indicate mean ± standard deviation (SD).

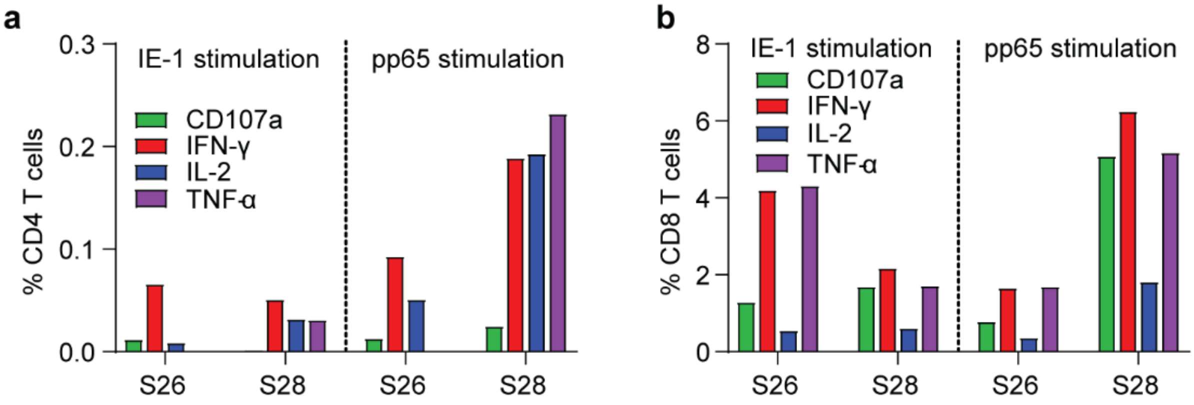

Supplementary Fig 3. Top 2 T-cell responders after 3-dose V160 vaccination. Percentages of IE-1 and pp65 responding (**a**) CD4 and (**b**) CD8 T cells that express four effector molecules (CD107a, IFN-γ, IL-2, and TNF-α) in month 9 PBMCs of subject 26 and 28 after background subtraction.

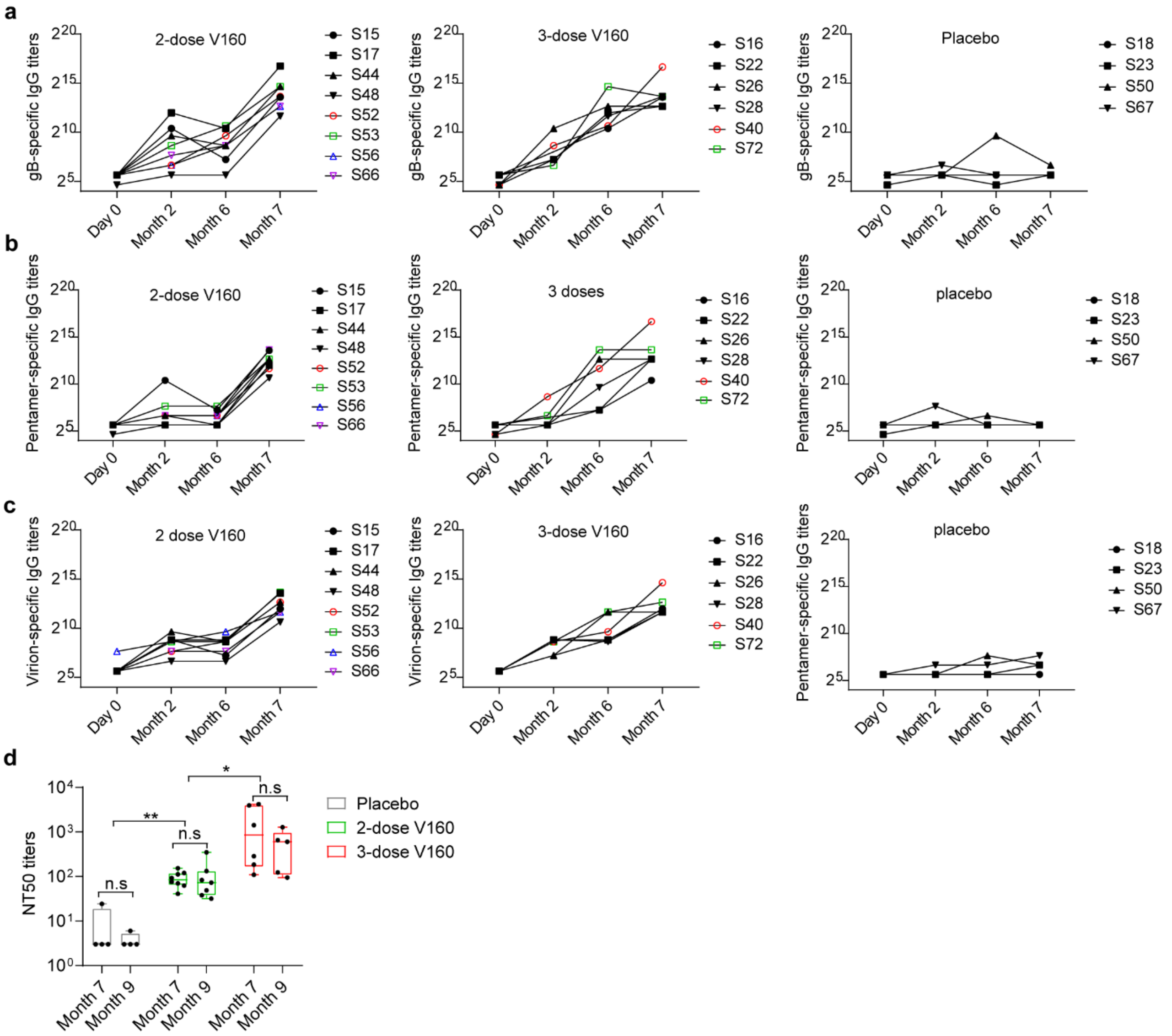

Supplementary Fig 4. Kinetics of antibody responses elicited by V160 vaccination. Serum samples available from 8 subjects in 2-dose V160 group, 6 subjects in 3-dose V160 group and 4 subjects in placebo group at day 1, month 2, month 6, and month 7 were determined for endpoint IgG titers for binding of (**a**) soluble gB, (**b**) soluble pentamer, and (**c**) HCMV virion by ELISA assay. **d**, The NT50 titers of month 7 and month 9 serum samples against HCMV strain AD169rev-GFP infection of ARPE-19 cells were determined. Data were plotted in a box and whiskers style showing median (center line), the first quartile, the third quartile together with all data points. Each dot represents one individual.

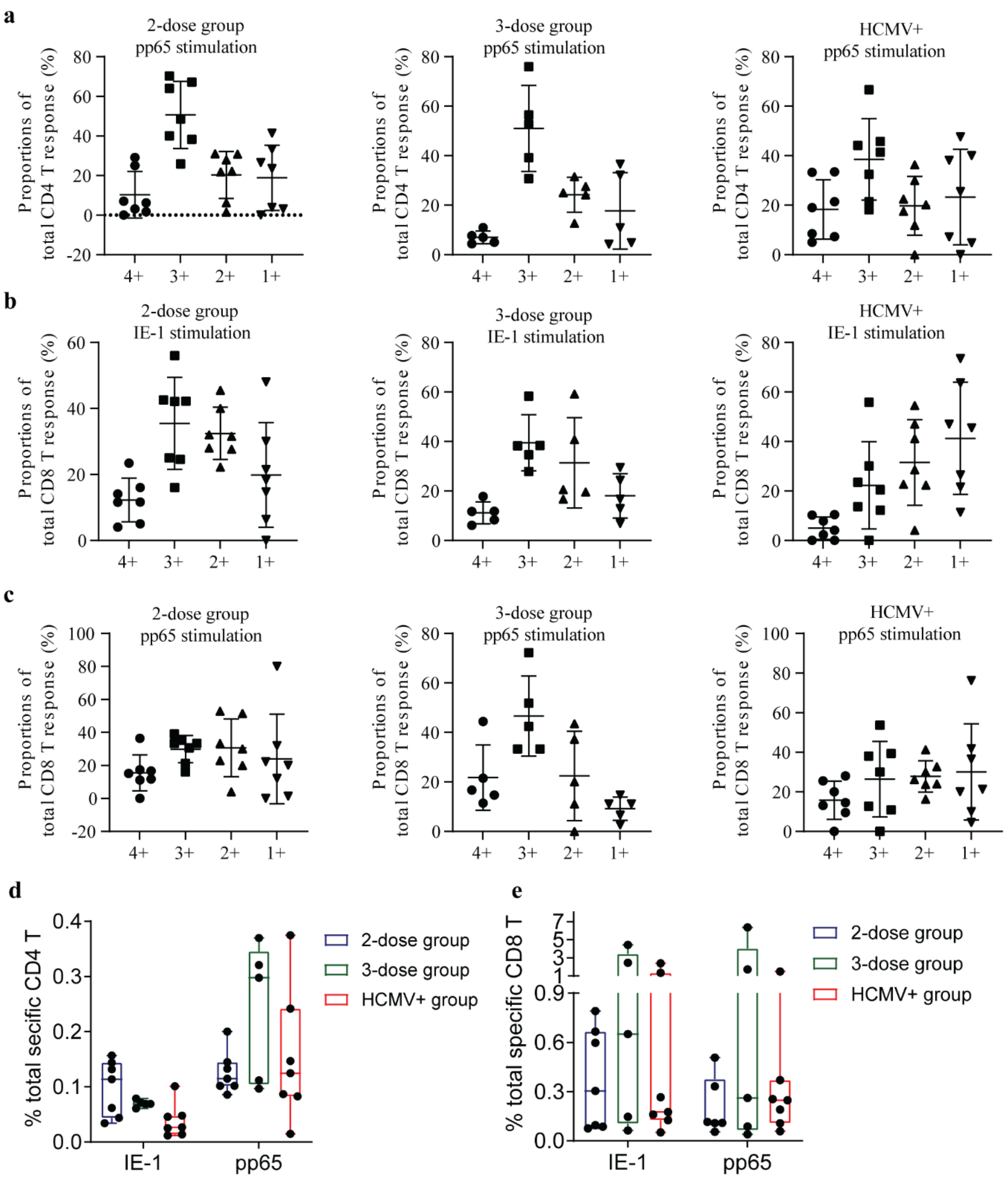

Supplementary Fig 5. Combinatorial analysis of T-cell responses in month 9 PBMCs of V160 subjects and HCMV positive donors. a, Proportions of total pp65 responsive CD4 T cells positive for 1, 2, 3, or 4 effector molecules (CD107a, IFN-γ, IL-2, and TNF-α) in three groups. **b-c,** Proportions of (**b**) IE-1 responsive and (**c**) pp65 responsive CD8 T cells positive for 1, 2, 3, or 4 effector molecules in three groups. Each symbol represents one individual. Bars indicate means ± SD. **d-e**, Percentages of total IE-1 and pp65 responsive (**d**) CD4 T cells and (**e**) CD8 T cells in three groups. Data were shown in a box and whiskers style showing median (center line), the first quartile, the third quartile together with all data points. Each dot represents one individual.

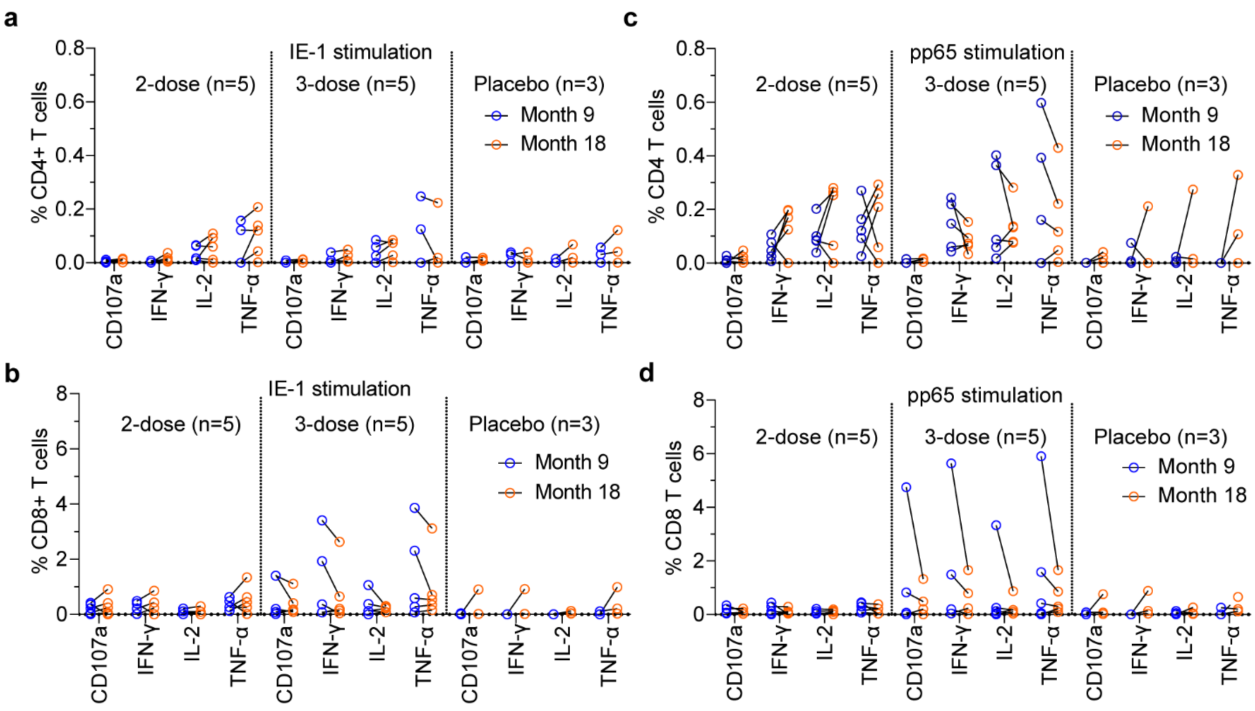

Supplementary Fig 6. Functional comparison of virus-specific T cells in month 9 and month 18 PBMCs. Month 9 and month 18 PBMCs that were available from the same 5 subjects in 2-dose group and 3-dose group, and the same 3 subjects in placebo group were analyzed side-by-side for IE-1 and pp65 specific T cell responses by ICS flow cytometry assay. **a, c**, The percentages of CD4 T cells that expressed each effector molecule (CD107a, IFN-γ, IL-2, and TNF-α) after IE-1 (**a**) and (**c**) pp65 stimulation were plotted, respectively. **b, d**, The percentages of CD8 T cells that expressed each effector molecule (CD107a, IFN-γ, IL-2, and TNF-α) after (**b**) IE-1 and (**d**) pp65 stimulation were plotted, respectively. All data were background subtracted in a sample matching manner. Each pair of connected circles represents the change of response from month 9 to month 18 in one individual.

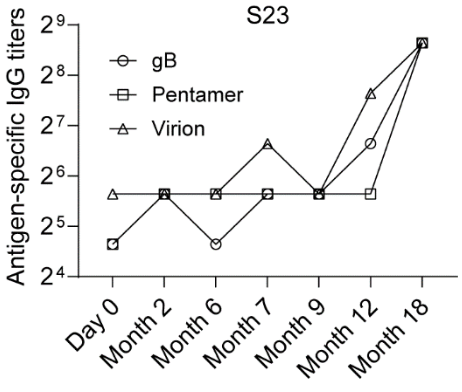

Supplementary Fig 7. Kinetics of HCMV specific antibody responses in subject 23. End-point titers of soluble gB, soluble pentamer and whole HCMV virion specific IgG antibodies in serum samples at indicated time points were determined by ELISA assay.

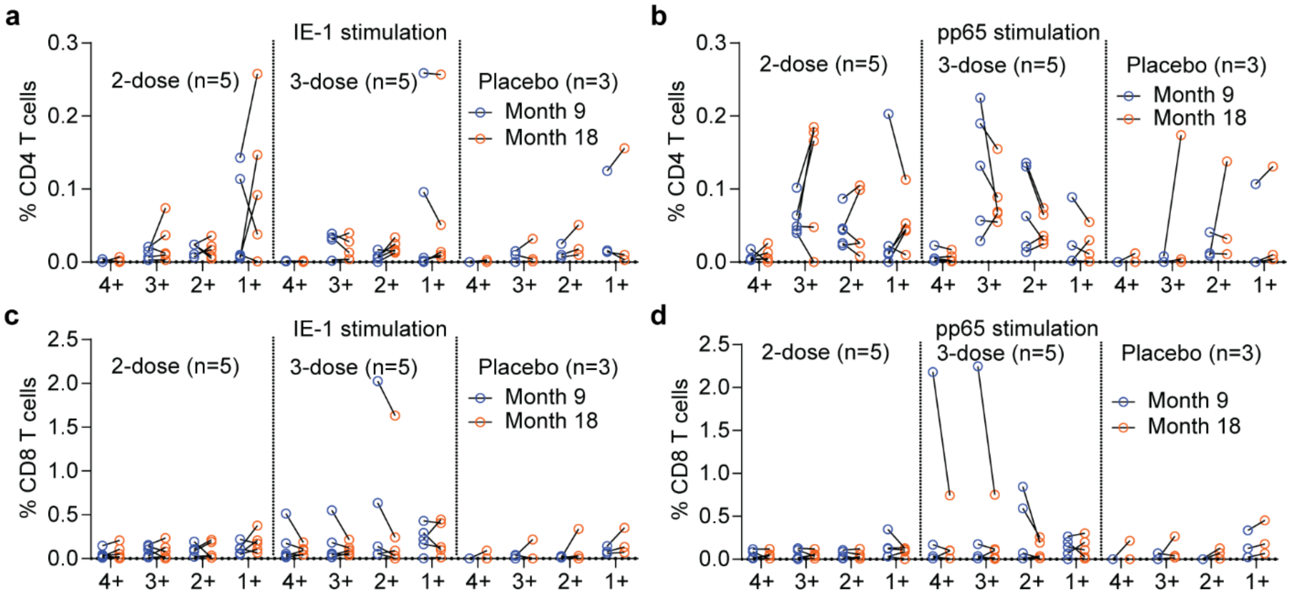

Supplementary Fig 8. Combinatorial analysis of T-cell responses at month 9 and month 18. The percentages of (**a-b**) CD4 T and (**c-d**) CD8 T cells that expressed 1, 2, 3 and 4 effector molecules (CD107a, IFN-γ, IL-2, and TNF-α) after (**a, c**) IE-1 and (**b, d**) pp65 stimulation in month 18 PBMCs (orange circle) were compared to those in month 9 PBMCs (blue circle). Available PBMCs samples from 5 subjects in 2-dose group, 5 subjects in 3-dose group, and 3 subjects in the placebo group were analyzed. All data were background subtracted in a sample matching manner. Each circle represents one individual.

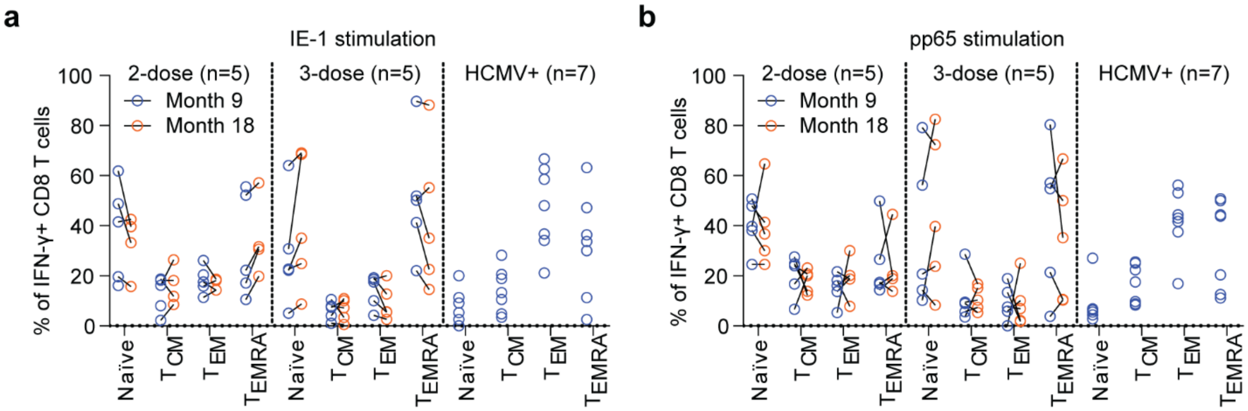

Supplementary Fig 9. Memory phenotypes of IFN-γ^+ CD8 T cells. Distributions of four memory phenotypes among (**a**) IE-1 and (**b**) pp65 responding IFN-γ^+ CD8 T cells in month 9 (blue circle) and month 18 PBMCs (orange circle), respectively. Data were from 5 subjects in 2-dose V160 group, 5 subjects in 3-dose V160 group, and PBMCs of seven HCMV$^+$ donors. Each circle represents one individual. Please be noted that M18 data of one 2-dose subject was absent due to none IFN-γ^+ CD8 T response, and the unusually high proportions of naïve pp65 responding CD8 T cells of two 3-dose subjects are probably caused by background interference due to low level responses. Percentages of individual specific T-cell responses were shown in Supplementary Fig 6.

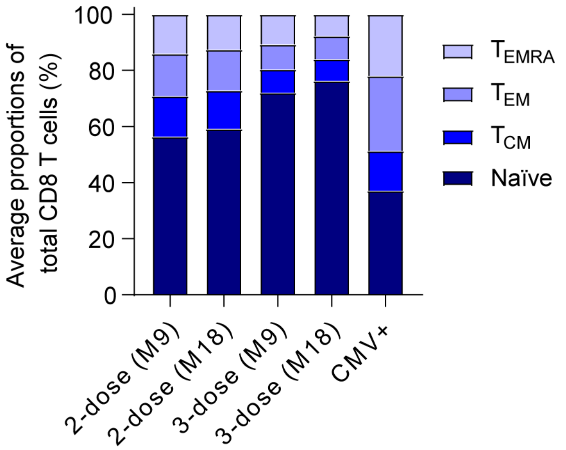

Supplementary Fig 10. Average proportions of four memory phenotypes among total CD8 T cells in 2-dose and 3-dose V160 subjects and seven HCMV$^+$ donors.
